# Design, synthesis, molecular docking and cytotoxic evaluation of novel pyrimidine-based sulfonamide derivatives as potent anticancer agents: SAR insights and biological profiling

**DOI:** 10.1038/s41598-026-41711-z

**Published:** 2026-03-24

**Authors:** Nesma M. Bayoumy, Ahmed A. Fadda, Hatem E. Gaffer, Nanees N. Soliman

**Affiliations:** 1 Dental Biomaterials Department, Faculty of Oral and Dental Medicine, Delta University for Science and Technology, Mansoura, Egypt, Mansoura, Egypt; 2 Department of Chemistry, Faculty of Science, Mansoura University, Mansoura, 35516 Egypt; 3 Department of Dyeing, Printing, and auxiliaries, National Research Centre, Textile institute, Giza, Cairo, Egypt

**Keywords:** Sulfonamide, Pyrazole, Thiazole, Pyrimidine, Anticancer Agents, Molecular Docking, Biochemistry, Cancer, Chemical biology, Chemistry, Computational biology and bioinformatics, Drug discovery

## Abstract

The low selectivity and significant toxicity of current chemotherapeutic medicines make the research and development of novel anticancer agents an urgent necessity. This study used IR, NMR, MS, and elemental studies to rationally design, synthesize, and structurally characterize a novel class of pyrimidine-based sulfonamide derivatives. A wide variety of heterocyclic scaffolds, such as oxadiazoles, thiazolinones, chromenes, pyrazoles, pyrazolopyrimidines, triazolopyrimidines, imidazolidines, and triazines, were made possible by the synthetic approach. HepG2, MCF-7, WI-38, and VERO cell lines were used to test the synthetic compounds’ cytotoxic properties. With IC_50_ values ranging from 7.4 to 10.2 µg/mL against HepG2 and 8.2 to 10.0 µg/mL against MCF-7, compounds **18**, **21**, **23**, and **24** showed extremely significant anticancer activity, nearly matching the potency of the reference medication 5-fluorouracil. Crucially, these substances showed a good therapeutic index with noticeably reduced toxicity toward normal WI-38 and VERO cells. The most active derivatives’ increased cytotoxicity and selectivity were shown to be largely due to electron-donating groups, balanced lipophilicity, and optimal heterocyclic substitution patterns, according to structure–activity relationship (SAR) research. The experimental results were corroborated by molecular docking experiments against thymidylate synthase (PDB: 2VF5), where compound **23** exhibited the highest binding affinity (S = − 5.5589 kcal/mol), creating strong H-bond interactions with Lys487 and Ala498 within the active region. Overall, compounds **18**, **21**, **23**, and **24** are identified as interesting lead candidates for additional development as effective and selective anticancer medicines based on the combined biology, SAR, and docking results. Future optimization, mechanistic research, and even in vivo assessment can all benefit from these discoveries.

**Supplementary Information:**

The online version contains supplementary material available at 10.1038/s41598-026-41711-z.

## Introduction

Despite significant progress in scientific and clinical research, cancer is still one of the most concerning diseases in the world^1^. Even though chemotherapy is the mainstay of cancer treatment, its use is often limited because of negative side effects, which increases the need for the creation of novel chemotherapeutic medications for more effective cancer therapies^2–4^.

Furthermore, the first medications to be widely and consistently used as chemotherapeutic and preventative agents against a variety of diseases were sulfonamides, sometimes known as sulfa drugs^5^. The antihypertensive medication bosentan^6^, antibacterial^7^, antiprotozoal^8^, antifungal^9^, anti-inflammatory^10^, nonpeptidic vasopressin receptor antagonists^11^, and translation initiation inhibitors^12^ are among the more than thirty medications with this functionality that are now in clinical use. Several significant sulfonamide compounds are employed as commercially significant carbonic anhydrase inhibitors^13^. Additionally, they are useful in treating scalds, ulcerative colitis^14^, rheumatoid arthritis^15^, male erectile dysfunction as the phosphodiesterase-5 inhibitor sildenafil—better known by its brand name, Viagra^16^ and obesity^17^. In more recent times, sulfonamides have been utilized to treat Alzheimer’s disease^18^, as an anticancer drug^19^, and as the antiviral HIV protease inhibitor amprenavir^20^.

**Fig. 1:**
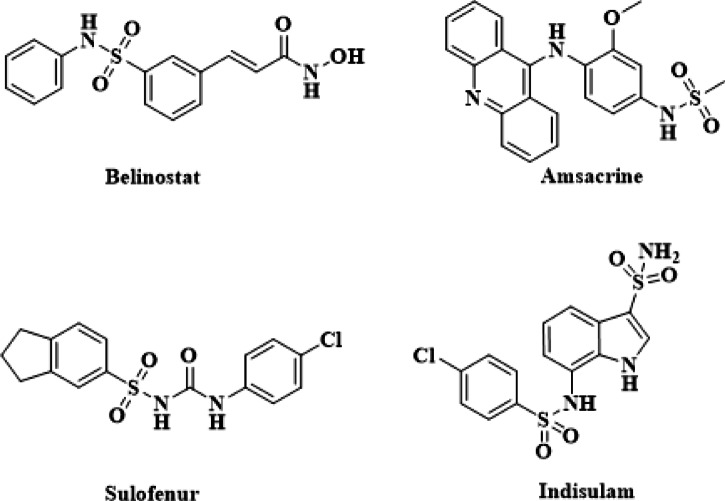
Anticancer drugs contain the secondary sulfonamide moiety.

Additionally, sulfonamide prodrugs were administered in inventions relating to pharmaceutical compositions for treating hyperproliferative disorders in mammals^21,22^. A focus of research on sulfonamides is due to their wide applications as inhibitors as well as their effects on tyrosine kinase, protein tyrosine phosphatase 1B, human immunodeficiency virus protease-1 (HIV-1), histone deacetylase, phosphatidyl inositol 3-kinase, angiogenesis, pyrazole kinase, sphingosine kinase, and tyrosyl DNA phosphodiesterase^23^. Hence, the sulfonamide moiety is present in the structures of various clinical drugs^1^, as carbonic anhydrase (CA) inhibitors (e.g., dorzolamide, brinzolamide (Azopt), and diclofenamide), diuretics (e.g., furosemide, indapamide, and chlorthalidone), cycloxygenase 2 (COX2) inhibitors (e.g., celecoxib and valdecoxib), and anti-cancer drugs (e.g., apricoxib and pazopanib)^24^. The FDA has approved some secondary sulfonamide derivatives for cancer treatment such as Belinostat, and Amsacrine **(**Fig. 1**)**^25,26^. Belinostat, a histone deacetylase (HDAC) inhibitor, has been licensed after Vorinostat and Romidepsin for the treatment of T-cell lymphoma. ABT-199, a Bcl-2 inhibitor with good selectivity, has been approved for the treatment of patients with chronic lymphocytic leukemia (CLL). Additionally, Amsacrine has been approved for the treatment of acute leukemias and malignant lymphomas by inserting itself between the DNA strands of tumor cells. Furthermore, many sulfonamide derivatives have been clinically tested for anticancer effects, including Sulofenur, and Indisulam (Fig. 1)^27^. Numerous sulfonamides have also been identified to inhibit CA, which allows them to operate as antitumor agents^28,29^. It has been demonstrated that aromatic or hetero-aromatic sulfonamides can counteract the effect of tumor acidification, which prevents the proliferation of cancer cells and inhibits tumor invasion mediated by CAs^30^. At the same time, pyrimidine derivatives and heterocyclic annulated pyrimidines have garnered a lot of attention due to their therapeutic properties^31–34^. These medicinal activities include anticancer^35^, antiviral^36^, and antitumor^37^. The pyrimidine ring is the building unit of RNA and DNA, and several pharmacological actions can be recognized in pyrimidine-based chemical structures. The anticancer activity of pyrimidines is one of their purported therapeutic properties that has received the most attention. The numerous mechanisms by which the pyrimidine-based scaffolds have exerted their effects suggest their ability to interact successfully with a variety of enzymes, targets, and receptors^38,39^. Moreover, reported studies recognized that the introduction of the sulfonamide group into the C-2 aniline scaffold of the pyrimidine template could enhance the activity against Bruton’s tyrosine kinase (BTK), which is a potent therapeutic target for treating B-cell lymphoblastic leukemia^40^. All of these observations inspired us to look into the synthesis of sulfonamides containing pyrimidine moieties and their anticancer potential. As a result, the inclusion of such a moiety is most likely to produce lead compounds of significant medical potency. Furthermore, a variety of pyrimidine sulfonamides containing chloro, phenol, and anilino substitutions were synthesized and tested for their anticancer activity. The activity of these new sulfonamide derivatives seemed encouraging and should be considered a possible lead for its development as a novel anticancer medication.

Based on the well-established anticancer relevance of sulfonamide pharmacophores and the pivotal role of pyrimidine scaffolds in nucleic acid metabolism and cancer therapy, a rational hybridization strategy was adopted in this study. The integration of sulfonamide and pyrimidine moieties was designed to generate multifunctional molecules capable of targeting key enzymes involved in cancer cell proliferation. Thymidylate synthase was selected as a molecular target due to its critical role in DNA synthesis and its established inhibition by pyrimidine-based drugs such as 5-fluorouracil. Accordingly, a series of structurally diverse pyrimidine-based sulfonamide derivatives were designed and synthesized to optimize biological activity and selectivity.

## Rational design strategy

The rational design of the synthesized compounds was based on a pharmacophore hybridization approach combining the sulfonamide moiety with a pyrimidine scaffold. Sulfonamides are well known for their strong hydrogen-bonding capacity and their presence in several clinically relevant anticancer agents, while pyrimidine derivatives mimic nucleobases and are associated with inhibition of key enzymes involved in DNA synthesis, particularly thymidylate synthase.

By integrating both pharmacophores into a single molecular framework, we aimed to generate multifunctional molecules capable of enhanced enzyme binding and improved anticancer activity. Structural diversification through heterocyclic annulation (e.g., triazole, pyrazole, triazine systems) was introduced to optimize electronic distribution, lipophilicity, and steric orientation, thereby improving cellular permeability and target interaction. This strategy was intended to enhance both potency and selectivity toward cancer cells while minimizing toxicity toward normal cells.

## Results and discussion

**Fig. 2:**
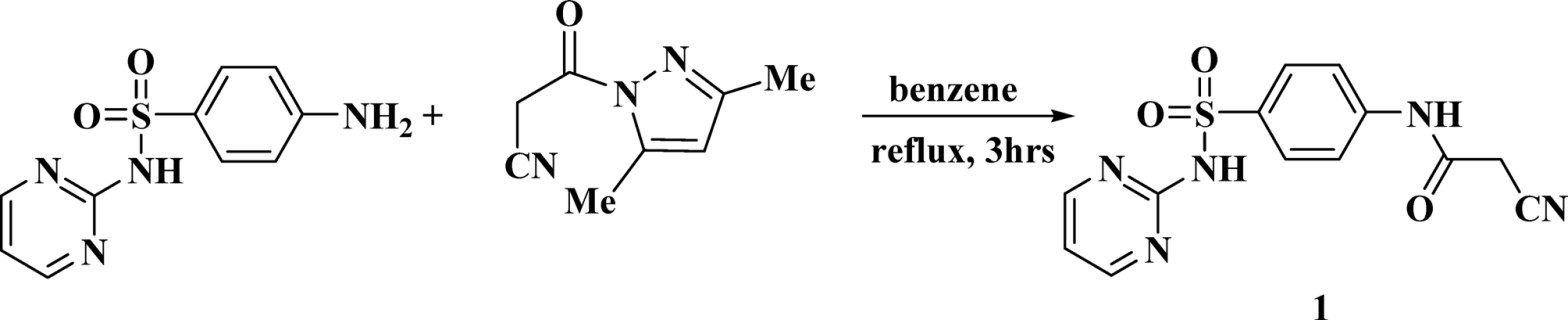
Synthesis of starting compound **1.**

**Fig. 3:**
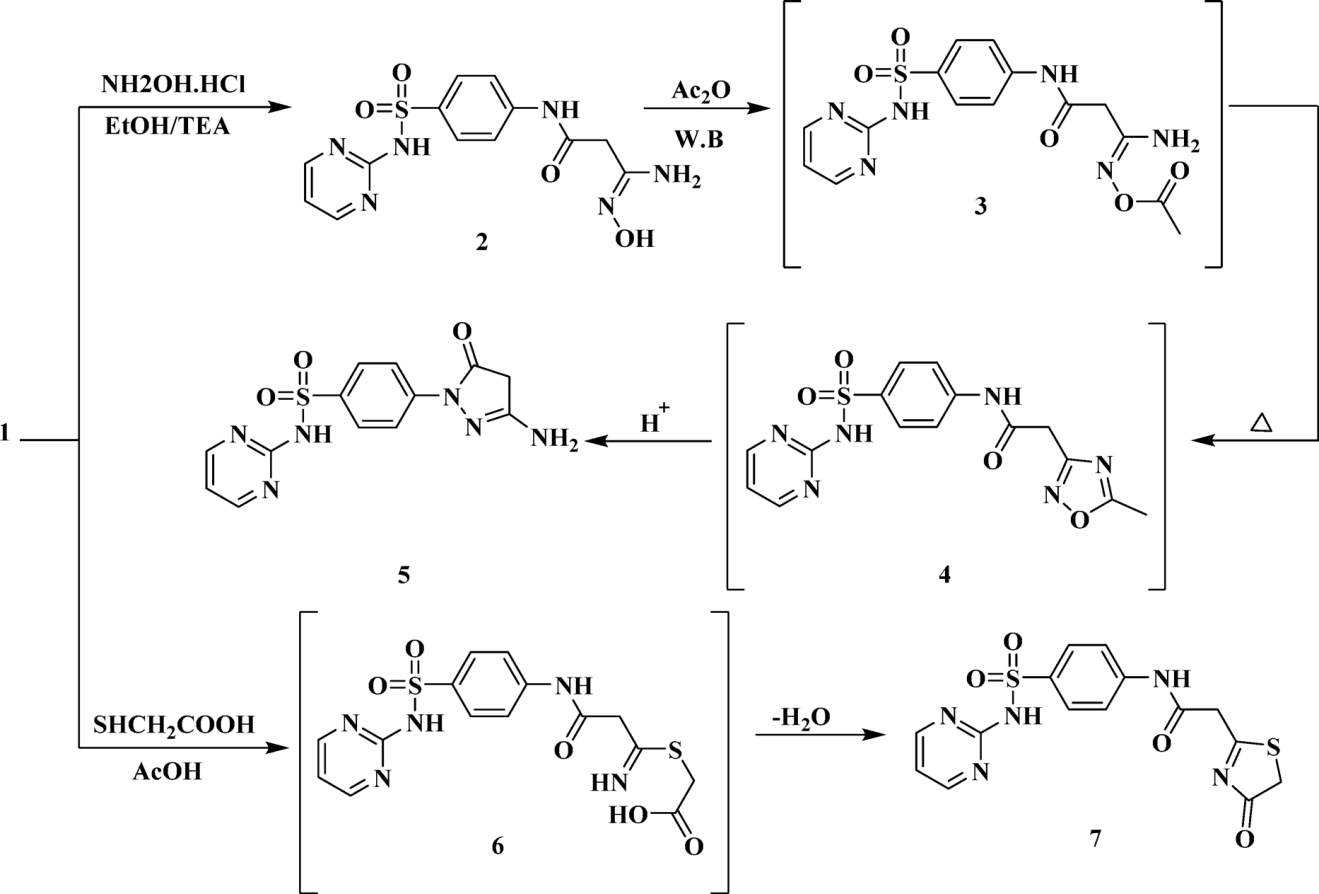
Reaction of cyanoacetyl sulfadiazine with hydroxyl amine hydrochloride and mercapto acetic acid.

**Fig. 4:**
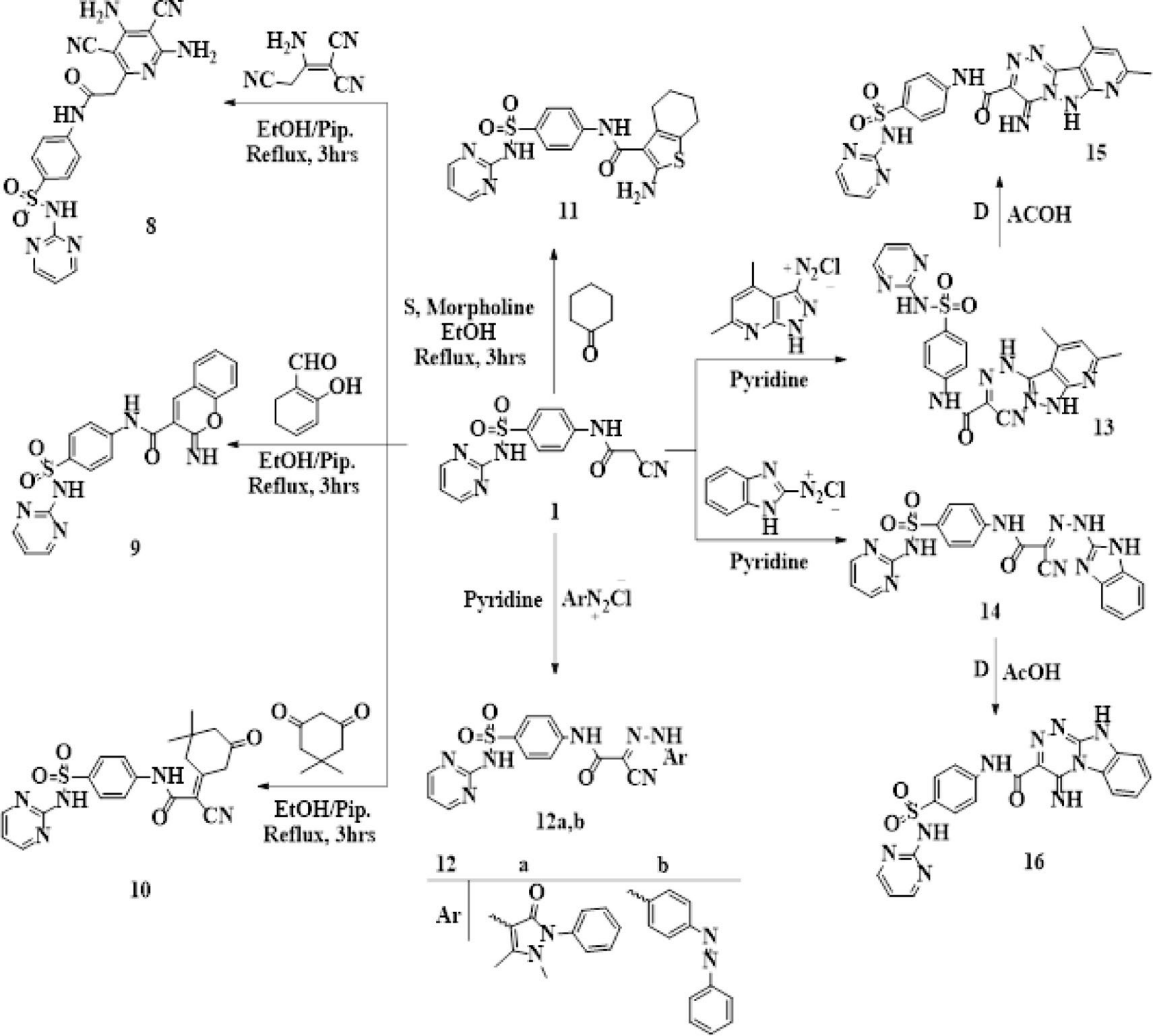
Reaction of cyanoacetyl sulfadiazine with different reagents.

**Fig. 5:**
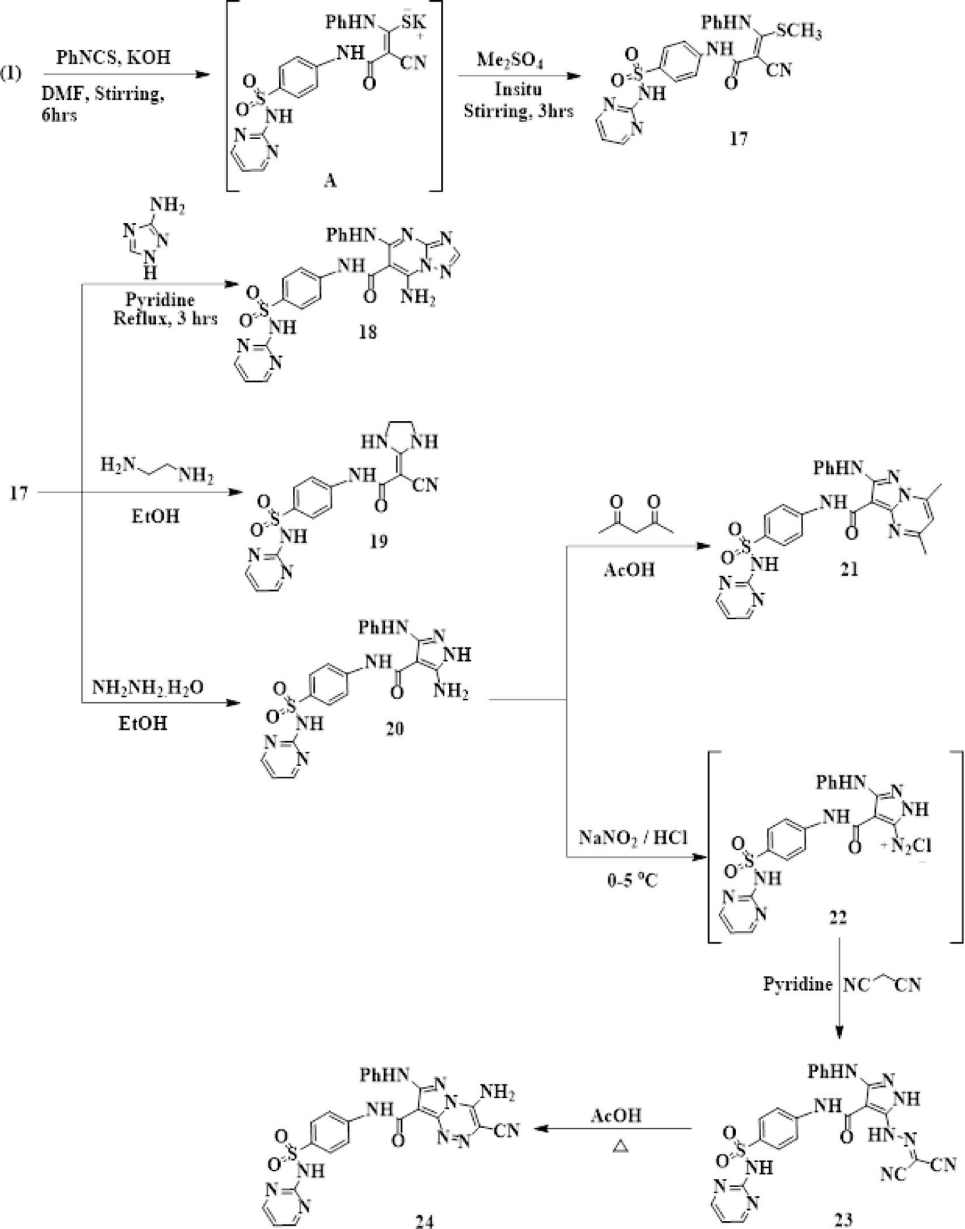
Reaction of thiocarbamoyl **17** with nitrogen N-nucleophiles and for mation of pyrazolotriazine derivative **24.**

Figures 2, 3, 4 and 5 describe the synthetic routes used to produce the target compounds. Using the previously described method^41^ (Fig. 2), cyanoacetylation of sulfadiazine in dry xylene with 3-(3,5-dimethyl-1 H-pyrazol-1-yl)-3-oxopropanenitrile produced the known important intermediate, 2-cyano-N-(4-(N-(pyrimidin-2-yl)sulfamoyl)phenyl)acetamide **(1)** in high yield^42^.

Amidoxime **2** was thus produced by treating precursor **1** with hydroxylamine in the presence of TEA as a base. *O*-acetylation was produced when amidoxime **2** was acetylated with acetic anhydride. Compound **3** was thermally cyclized to get 1,2,4-oxadiazole derivative **4**, which was then rearranged by acid catalysis to yield pyrazolin-5-one derivative **5** (Fig. 3). The absence of nitrile function in the amidoxime **2’s** infrared spectrum, the emergence of a hydroxyl group at absorption band 3510 cm^− 1^, and amino and imino function at absorption bands 3430, 3230 and 3210 cm^− 1^ were its defining characteristics. The structure of **5** was thus identified by the presence of a singlet due to two protons at δ 3.19 ppm in the ^1^H-NMR spectrum, which represents the methylene protons of the pyrazolone moiety; two doublets attributed to the benzene ring at δ 7.84, 8.25 ppm, which were coupled to each other with coupling constant, *J* = 4.4 Hz; a multiplet signal at δ 7.10–7.20 ppm assigned to the C_5_H-pyrimidine proton; and a singlet signal at δ 11.27 ppm due to the *N*HSO_2_ proton. Additionally, stretching frequencies of 3410, 3210, 1680, and 1310 cm^− 1^ were detected in its infrared spectra and attributed to NH_2_, NH, amidic CO, and SO_2_, respectively.

The thiazolinone derivative **7** was produced in large quantities by cyclocondensation of cyanoacetanilide **1** with 2-mercapto acetic acid in boiling glacial acetic acid. Thiazolinone derivative **7’s** infrared spectra showed stretching frequencies at 3290 and 3250 cm^− 1^, which were attributed to 2NH groups, and a significant absorption band at 1680 and 1675 cm^− 1^, which were assigned to 2CO functions. Its ^1^H-NMR spectra showed a singlet signal at δ 3.10 ppm attributed to methylene protons and a singlet signal at δ 4.04 ppm corresponding to two protons attributed to methylene protons of the thiazolinone moiety.

Here, we reported the reaction of cyanoacetanilide **1** reacts with malononitrile dimer in refluxing EtOH with a few drops of piperidine to produce pyridinyl acetanilide **8**. The spectral measurements of structure 8 were consistent. The goal of 2-iminochromene derivative **9** was achieved by reacting compound **1** with salicylaldehyde in hot ethanol with catalytic drops of piperidine (Fig. 4). Conversely, compound **10** was synthesized when compound **1** interacted with dimedone while hot ethanolic piperidine was present **(**Fig. 4).

Additionally, the required thiophene derivative **11** was produced under the Gewald reaction conditions by heterocyclization of cyanoacetanilide **1** with both cyclohexanone and elemental sulfur in ethanol upon heating under reflux and the presence of drops of catalytically morpholine (basic catalyst) (Fig. 4). IR, ^1^H-NMR, and elemental studies all support the suggested structure. Additionally, compound **1** diazocoupled with antipyrine diazonium chloride and azobenzene diazonium chloride in pyridine at 0–5 °C to produce the expected highly physiologically active hydrazone derivatives **12a** and **12b**, respectively (Fig. 4). The presence of two singlet signals equivalent to two methyl protons at δ 2.25, 3.15 ppm in the ^1^H-NMR spectrum, which indicate the CH_3_, N-CH_3_ protons of the pyrazolone moiety of antipyrine, confirmed sulfonamide pyrimidine containing antipyrine nucleus, **12a**. The IR spectra of **12a** showed stretching frequencies at 3330 − 3255, 2218, 1680, and 1675 cm^− 1^ that might be attributed to 3NH, nitrile, and two amidic carbonyls functions. The infrared spectra of **12b** showed absorption bands at 3345 and 3260 cm^− 1^ that were attributed to (3NH) functions, an absorption band at 2220 cm^− 1^ that was attributed to the (CN) function, and an absorption band at 1677 cm^− 1^ that was attributed to the amidic (CO) function group.

The synthesis of bridged-head nitrogen heterocyclic systems from diazotized heterocyclic amines, a perfect building block, is described in this work. The corresponding hydrazono compounds **13** and **14** were therefore obtained by coupling the crucial intermediate **1** with 4,6-dimethyl-1 H-pyrazolo[3,4-b]pyridin-3-diazonium chloride and 1 H-benzo[d]imidazol-2-diazonium chloride in pyridine at 0–5 °C. Compounds **13** and **14** cyclized to compounds **15** and **16**, respectively, when heated in refluxing acetic acid (Fig. 4).

In addition to three singlets at δ 2.61, 2.68, and 6.87 ppm characterized for two CH_3_ protons of the pyridine ring and one aromatic proton of the pyridine ring H-3, the ^1^H-NMR spectrum of **15** showed three D_2_O-exchangeable singlets at δ 8.15, 9.34, 9.99, and 11.36 ppm due to endo cyclic NH, NHCO, =NHSO_2_ protons. The absence of a cyano group absorption band was shown in the IR spectra of **16**. The amidic carbonyl group showed a high absorption at 1685 cm^− 1^, whereas four NH showed a broad absorption band at 3310–3260 cm^− 1^.

The non-isolable intermediate thiocarbamoyl salt (A) was produced when **1** was reacted with phenyl isothiocyanate in DMF with potassium hydroxide at room temperature. The equivalent acrylamide **17** was produced when the intermediate thiocarbamoyl salt A was insitu alkylated with (CH_3_)_2_SO_4_ (Fig. 5). Spectral and elemental investigations confirmed the presence of the ketene N, S-acetal **17**. A singlet signal at δ 2.30 ppm in its ^1^H-NMR spectrum was attributed to SCH_3_ protons. Acrylamide **17’s** reactivity with nitrogen nucleophiles was examined. Triazolo[1,5-a]pyrimidine derivative **18** was then obtained by heating compound **17** with 3-amino-1H-1,2,4-triazole in pyridine. The Michael addition of the amino group to the ethylenic bond in **17**, followed by the removal of methanethiol, could be assumed as the reaction mechanism. The nitrogen atom was then nucleophilically added to the nitrile carbon to yield **18** (Fig. 5). Its spectral measurements supported Structure 18. As a result, its infrared spectrum showed absorption bands at 3440, 3310 − 3260, and 1681 cm^− 1^ that were attributed to NH_2_, 3NH, and amidic CO functions, respectively, and showed no conjugated cyano function absorption band.

The imidazolidine derivative **19** was produced by treating **17** with bifunctional nucleophilic reagents, such as ethylenediamine in boiling ethanol (Fig. 5). In addition to a significant absorption band at 1670 cm^− 1^ attributable to the carbonyl function, the IR spectra of **19** revealed stretching bands at 3330 –3260 cm^− 1^ attributed to four NH groups and an absorption band at 2220 cm^− 1^ related to the cyano function. The two methylene protons were responsible for the signal at δ 3.44 ppm in its ^1^H-NMR spectrum, which is equivalent to four protons. The required 5-aminopyrazole derivatives **20** were produced by cyclocondensation of acrylamide **17** with hydrazine hydrate in EtOH after heating under reflux. The IR spectra of **20** showed that the amidic (CO) function group is responsible for the absorption band at 1687 cm^− 1^, the (4NH) groups are responsible for the absorption band at 3275 cm^− 1^, and the (NH_2_) function is responsible for the absorption band at 3460 cm^− 1^. NH_2_ protons were identified by ^1^H-NMR as a singlet signal equivalent for two protons at δ 6.05 ppm.

5-Aminopyrazoles have been widely employed as a crucial intermediary for the synthesis of many polyfunctionalized fused pyrazoles with expected biological response and as a flexible precursor for the synthesis of a variety of heterocycles. Pyrazolo[1,5-*a*]pyrimidine derivative **21** was therefore produced by the cyclocondensation reaction of aminopyrazole 20 with acetylacetone in glacial acetic acid when heated under reflux (Fig. 5). With a parent ion peak (M^+^) at m/z 514, attributed to the chemical formula C_25_H_22_N_8_O_3_S, the reported structure 21 was consistent with its IR, NMR, and elemental analysis. Additionally, compound **20** was diazotized with sodium nitrite and concentrated HCl to produce the matching diazonium chloride **22**. This was then coupled with malononitrile in pyridine to produce the intended hydrazono derivatives **23**. Pyrazolo[5,1-c]^1,2,4^ triazine derivatives **24** were obtained by heating compound **23** in glacial acetic acid (Fig. 5). The IR spectra of **23** showed a carbonyl function at 1702 cm^− 1^, two cyano functions at 2219 and 2198 cm^− 1^, large absorption bands at 3340 and 3313 cm^− 1^ attributable to (5NH), and an azo peak at 1573 cm^− 1^.

### Cytotoxicity evaluation

The cytotoxic evaluation of the newly synthesized compounds against HepG2, WI-38, VERO, and MCF-7 cell lines revealed significant differences in their anticancer potential and selectivity profiles (Table 1). Overall, several compounds exhibited potent inhibitory effects toward cancer cells while maintaining relatively low toxicity against normal cell lines, indicating a favorable therapeutic window.

**Table 1:** Cytotoxic evaluation of the newly synthesized compounds (IC_50_ µM).

Compound	HepG2	WI-38	VERO	MCF-7
**2**	227.0 ± 0.36	253.0 ± 0.03	260.0 ± 0.19	217.0 ± 0.05
**5**	199.5 ± 0.33	235.3 ± 0.31	224.0 ± 0.01	208.0 ± 0.01
**7**	150.7 ± 0.21	187.0 ± 0.04	177.6 ± 0.11	142.0 ± 0.13
**8**	64.7 ± 0.22	123.3 ± 0.16	113.7 ± 0.18	73.7 ± 0.17
**9**	76.6 ± 0.02	140.5 ± 0.22	119.6 ± 0.23	88.3 ± 0.32
**10**	128.1 ± 0.23	151.3 ± 0.36	128.1 ± 0.05	135.6 ± 0.23
**11**	66.6 ± 0.33	112.5 ± 0.21	85.2 ± 0.03	60.8 ± 0.14
**12a**	44.2 ± 0.17	77.1 ± 0.35	72.6 ± 0.02	43.8 ± 0.18
**12b**	47.0 ± 0.33	97.4 ± 0.14	88.3 ± 0.14	46.0 ± 0.13
**13**	27.5 ± 0.06	84.0 ± 0.23	75.6 ± 0.11	32.8 ± 0.11
**14**	36.0 ± 0.11	96.0 ± 0.44	85.2 ± 0.31	41.6 ± 0.09
**15**	26.7 ± 0.16	78.7 ± 0.11	70.5 ± 0.07	30.8 ± 0.02
**16**	36.4 ± 0.14	67.8 ± 0.16	72.2 ± 0.02	46.0 ± 0.03
**17**	34.5 ± 0.13	77.4 ± 0.23	79.7 ± 0.18	39.0 ± 0.01
**18**	18.0 ± 0.03	40.0 ± 0.13	58.7 ± 0.04	16.3 ± 0.03
**19**	177.2 ± 0.23	174.6 ± 0.34	180.3 ± 0.22	187.0 ± 0.07
**20**	54.6 ± 0.15	114.1 ± 0.29	107.9 ± 0.09	102.3 ± 0.02
**21**	19.8 ± 0.02	45.3 ± 0.13	56.9 ± 0.07	17.9 ± 0.18
**23**	15.9 ± 0.03	40.2 ± 0.01	45.7 ± 0.01	18.0 ± 0.07
**24**	14.0 ± 0.01	39.0 ± 0.21	47.6 ± 0.03	18.9 ± 0.03
**5-FU**	66.1 ± 0.03	24.6 ± 0.01	50.0 ± 0.04	17.7 ± 0.02

HepG2 liver cancer cells demonstrated the highest sensitivity to the tested compounds. Compounds **24**,** 23**,** 18**, and**21** showed **very strong cytotoxic activity**, with IC₅₀ values of **14.0**, **15.9**, **18.0**, and **19.8 µM**, respectively. These values are comparable to or slightly higher than the reference drug **5-fluorouracil (5-FU**,** IC₅₀ = 66.1 µM)**. Compounds **13**,** 15**,** 16**, and **17** also demonstrated strong activity, with IC₅₀ values ranging from 27.5 to 36.4 µM. In contrast, compounds such as **2**,** 5**, and **19** exhibited weak cytotoxicity, indicating lower affinity or limited cellular uptake. These results highlight compounds **24**, **23**, **18** and **21** as the most promising candidates for liver cancer inhibition.

A similar pattern was observed for the MCF-7 cell line, where compounds **18**,** 21**,** 23**, and **24** again showed **very strong cytotoxic activity**, with IC₅₀ values between **16.3 and 18.9 µM**. Although 5-FU displayed higher potency (IC₅₀ = 17.7 µM), the newly synthesized compounds still demonstrated significant activity. Moderate activity was observed for compounds **7**, **9**, **10**, and **20**, whereas compounds **2** and **5** showed weaker inhibitory effects. Taken together, compounds **18**, **21**, **23**, and **24** consistently constitute the most active series against both liver and breast cancer cells.

Assessment of cytotoxicity toward normal cells is critical in determining the safety and selectivity of anticancer agents. The majority of the tested compounds exhibited moderate to weak toxicity against WI-38 and VERO cells. Notably, compounds **18**,** 21**,** 23**, and**24**, despite their strong anticancer activity, maintained moderate IC₅₀ values (39-58.7 µM) toward WI-38 and VERO cells, indicating acceptable safety margins. In contrast, 5-FU demonstrated **marked toxicity** against normal cells, particularly WI-38 (IC₅₀ = 24.6 µM), confirming its well-known lack of selectivity. The lower toxicity of the synthesized compounds suggests a more favorable therapeutic profile.

### Structure–activity relationship (SAR) considerations

Clear SAR trends can be inferred from the data. Compounds belonging to the **18–24 series** consistently demonstrated enhanced cytotoxicity, suggesting that their structural features—such as increased lipophilicity, optimized ring substitution, or reduced steric hindrance—may facilitate improved cellular penetration or stronger interaction with biological targets. Conversely, compounds with higher IC₅₀ values, including **2**,** 5**,** and 19**, may lack optimal substituent orientation or electronic characteristics needed for effective activity. These observations provide valuable insight for future structural optimization.

### Selectivity and therapeutic potential

Selectivity is a key criterion in anticancer drug development. Compounds **18**,** 21**,** 23**,** and 24** displayed **high selectivity indices**, characterized by strong activity toward cancer cells and significantly lower toxicity toward normal cells. This contrasts with 5-FU, which, despite its potent anticancer activity, was highly cytotoxic to normal fibroblasts. The improved selectivity of the newly synthesized compounds underscores their potential as safer and more effective anticancer agents.

In summary, the cytotoxicity results highlight compounds **18**, **21**, **23**, and **24** as the most promising candidates, exhibiting very strong activity against HepG2 and MCF-7 cancer cell lines while retaining acceptable safety toward normal cells. Their superior selectivity compared with 5-FU indicates significant therapeutic potential and warrants further investigation through mechanistic, molecular docking, and in vivo studies.

### Structure–activity relationship (SAR) analysis

The cytotoxicity data presented in Table 1 allow the establishment of clear structure–activity relationship (SAR) trends among the synthesized compounds. Several structural features appear to strongly influence the potency and selectivity of the compounds toward cancer cell lines.

The most active compounds (**18**, **21**, **23**, and **24**) share common structural features that likely contribute to their superior activity:

They contain electron-donating substituents, which enhance electron density across the pharmacophoric scaffold. These groups may facilitate stronger interactions with cellular targets involved in apoptosis or cell-cycle regulation. The enhanced electron density may also improve molecular recognition and binding affinity.

In contrast, compounds **2**, **5**, and **19**, which exhibited weak activity, appear to lack these favorable electronic features, suggesting that electron-withdrawing or sterically demanding substituents negatively impact cytotoxic potency.

Compounds displaying high potency consistently share moderate to high lipophilicity, which improves:

- Cell membrane permeability,
- Intracellular accumulation, and.
- Target accessibility.

Compounds **18**, **21**, **23**, and **24** likely benefit from an optimal lipophilic balance that promotes penetration into cancer cells without excessively increasing nonspecific toxicity. Less active analogues (e.g., compounds **2** and **19**) may be too hydrophilic or may possess bulky substituents that limit membrane diffusion.

The most potent compounds tend to exhibit reduced steric hindrance around the active core. Excessive bulk near the pharmacophore may:

- Obstruct interactions with critical binding sites,
- Decrease binding affinity,
- Impair proper orientation within the target protein.

Therefore, the superior activity of compounds **18–24** suggests that their steric profile supports optimal molecular fitting into the target binding domain.

The compounds that showed the highest cytotoxicity share specific ring substitution patterns that likely enhance both electronic distribution and conformational flexibility. These substitution patterns may favor:

- Improved π–π stacking or hydrophobic interactions,
- Enhanced planarity of the molecule,
- Stronger binding to key enzymes or DNA structures involved in cancer cell proliferation.

Compounds with suboptimal substitution patterns (compounds **2**, **5**, **7**, and **19**) demonstrated significantly weaker activity, indicating that proper ring decoration is critical for activity.

An important SAR observation is the improved selectivity of compounds **18**,** 21**,** 23**, and **24**. Their structural characteristics not only enhance cytotoxicity toward cancer cells but also minimize toxicity toward normal cell lines. This suggests:

- A possible target-specific mechanism,
- Better differential uptake by cancer cells driven by structural advantages,
- Lower nonspecific interactions with normal cellular components.

This distinction highlights the pivotal role of optimized substituents in controlling both potency and safety.

### Molecular docking

**Fig. 6:**
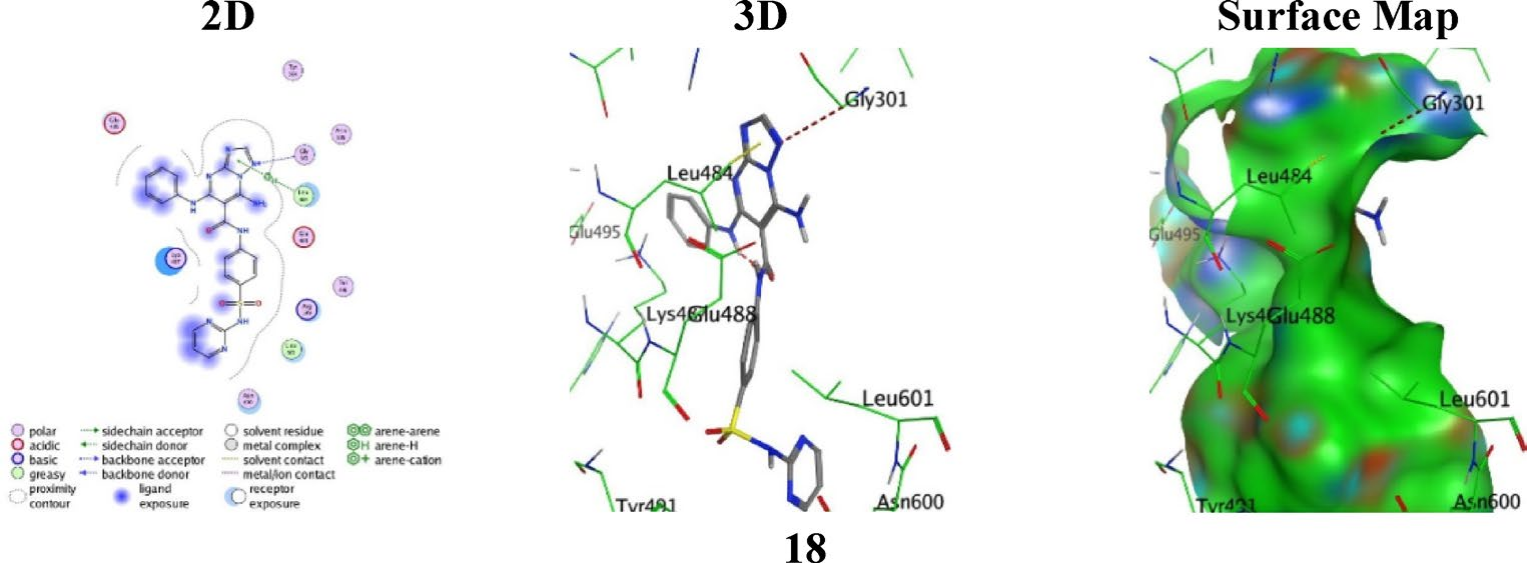
the molecular docking images between the analogue **18** and PDB: 2VF5.

The docking scores for the selected analogues **18**, **21**, **23**, and **24** toward the thymidylate synthase protein (PDB: 2VF5) are with good bindings compared to the reference 5-fluorouracil, indicating high compatibility with the active pocket of the enzyme protein (Table 2). Specifically, analogue **18** recorded a binding score (S = -5.2198 kcal/mol and RMSD = 1.4076 Å), which is stabilized by one accepting hydrogen-bonding among the N10 atom of the triazole-ring and Gly301 (3.64 Å), complemented by a π–H stacking with Leu484 (4.47 Å) revealing diverse polar and hydrophobic anchoring throughout the binding groove (Fig. 6).

**Fig. 7:**
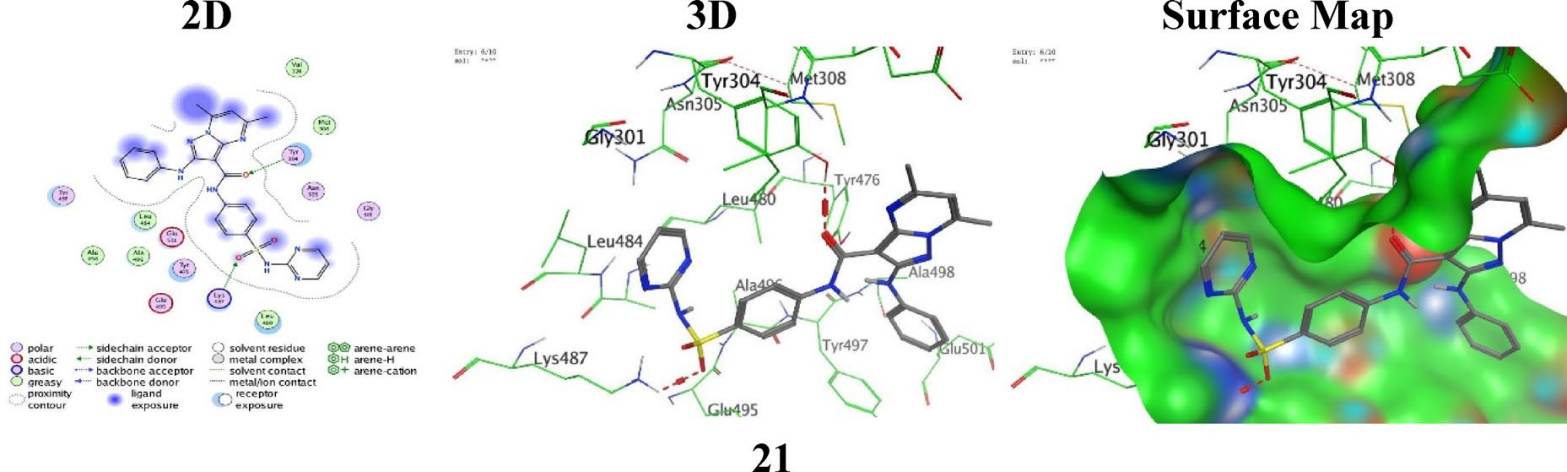
the molecular docking images between the analogue **21** and PDB: 2VF5.

Analogue **21** showed an improved binding score (S = -5.3020 kcal/mol and RMSD = 1.9988), forming two strong accepting hydrogen bindings: one between the oxygen O3 atom of the amide moiety with Tyr304 (3.11 Å) and the oxygen O12 atom of the sulphonamide group with Lys487 (2.95 Å), creating a wide polar anchoring across both the aromatic and the basic residues and drawing deeper into the amino-acid pocket (Fig. 7).

**Fig. 8:**
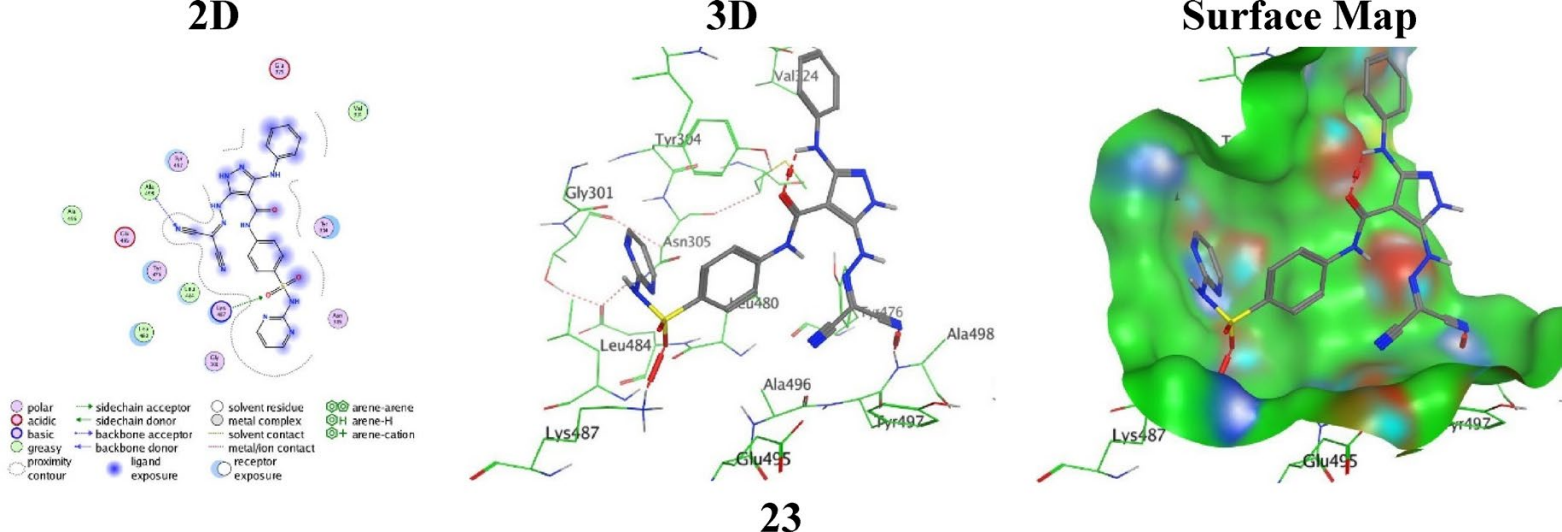
the molecular docking images between the analogue **23** and PDB: 2VF5.

The highest binding analogue, **23**, reached the highest binding affinity with a binding score ( S = -5.5589 kcal/mol with RMSD = 1.8323 Å), driven by two strong acceptor H-bonds: one between the oxygen O12 atom of the sulphonamide group with Lys487 at an optimal distance = 2.87 Å, and another between the N38 atom of the nitrile moiety with Ala498 at distance = 3.27 Å, resulting in dual anchoring across the pocket that maximizes electrostatic complementarity, thus stabilizing ligand alignment (Fig. 8).

**Fig. 9:**
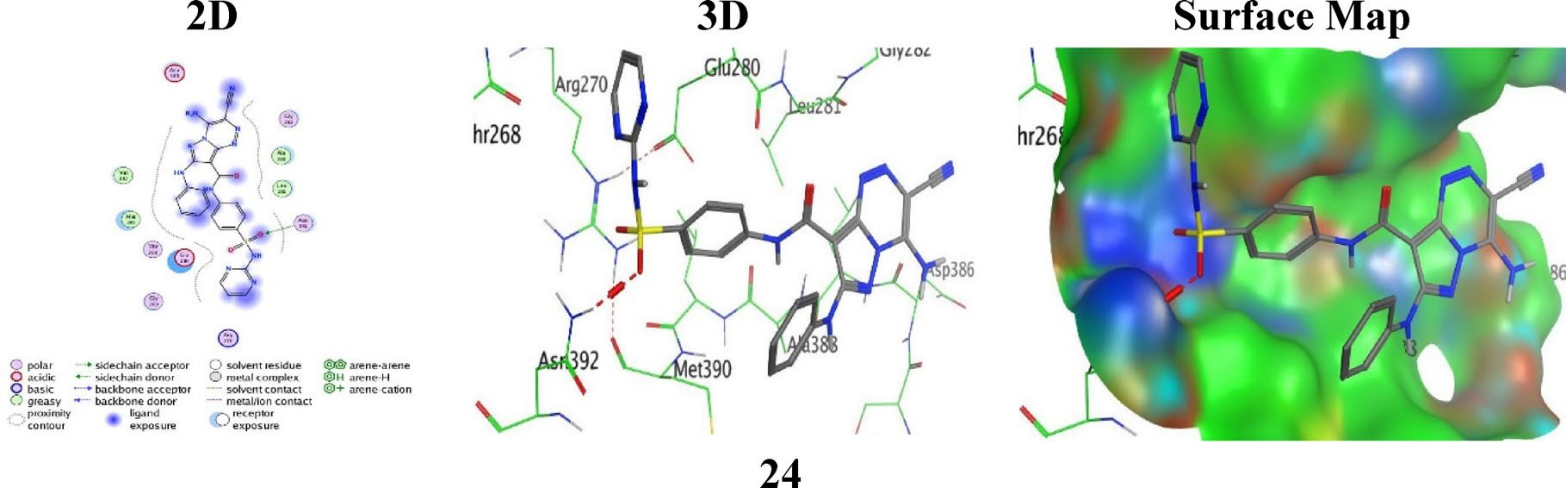
the molecular docking images between the analogue **24** and PDB: 2VF5.

Analogue **24**, in spite of fewer contacts, still showed a strong binding energy (S = -5.0656 kcal/mol and the lowest RMSD = 1.2156 Å) that proposes a highly stable pose maintained mainly by a H-bonding acceptor between the oxygen O13 atom of the sulphonamide group and Asn392 (3.07 Å) (Fig. 9).

**Fig. 10:**
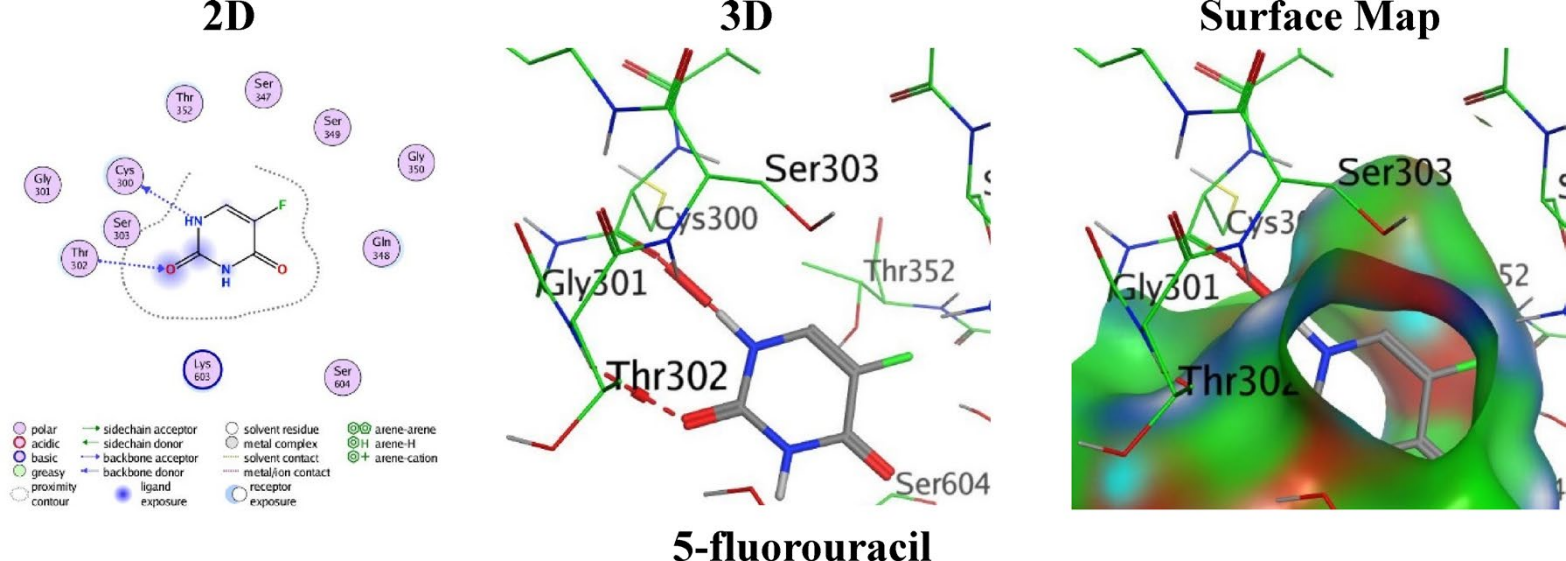
the molecular docking images between **5-fluorouracil** and PDB: 2VF5.

For comparison, the standard drug 5-fluorouracil demonstrated a weak score (S = -3.7870 kcal/mol), depend on only two moderate bindings: an H-donor bond between N1atom of the pyrimidinedione ring and Cys300 (2.89 Å) and an H-acceptor bond between O3 of the oxo group and Thr302 (3.35 Å). Co-operatively, these results recommend analogue **23** as the most promising inhibitor scaffold, followed by analogues **21** and **18** (Fig. 10).

**Table 2:** Molecular docking results between the potent compounds and PDB: 2VF5.

No	Binding energy (S kcal/mol)	RMSD	Ligands and amino acids interaction	Bond type	Distances (A°)
18	− 5.2198	1.4076	N 10 of the triazole-ring with Gly301 The triazole-ring with Leu484	H-acceptor pi-H	3.64 4.47
21	− 5.3020	1.9988	O 3 of the amide group with Tyr304 O 12 of the sulphonamide group with Lys487	H-acceptor H-acceptor	3.11 2.95
23	− 5.5589	1.8323	O 12 of the sulphonamide group with Lys487 N 38 of the nitrile group with Ala498	H-acceptor H-acceptor	2.87 3.27
24	− 5.0656	1.2156	O 13 of the sulphonamide group with Asn392	H-acceptor	3.07
5-fluorouracil	− 3.7870	1.6530	N 1 of the pyrimidinedione ring with Cys300 O 3 of the oxo group with Thr302	H-donor H-acceptor	2.89 3.35

It should be emphasized that molecular docking was used to provide a mechanistic hypothesis for the observed anticancer activity rather than definitive proof of thymidylate synthase inhibition. Further enzymatic and cell-cycle/apoptosis studies are planned to validate this mechanism.

Given the pyrimidine-based structure of the synthesized compounds and their strong activity against rapidly proliferating HepG2 and MCF-7 cells, thymidylate synthase represents a biologically relevant and rational target for initial in-silico evaluation.

## Experiment

### Synthesis and procedure

See supplementary File.

### Antitumor evaluation

The cell lines HepG-2, WI38, VERO, and MCF-7 were obtained from the American Type Culture Collection (ATCC).

It was carried out according to the previously reported work^43^.

### Molecular Docking

Using the Protein Data Bank (PDB) (https://www.rcsb.org), the crystal structures of thymidylate synthase binding site represented by (ID Code: 2VF5) were downloaded^44^. Compounds **18**, **21**, **23**, and **24**’s chemical structures were created using the ChemDraw 16.0 program, which was ascribed with the proper 2D, 3D, and surface map positioning. Besides, ChemBio3D program was used to minimize the potential energy of each individual compound. To create the ligands and maintain them in PDB format, the goal protein was first converted in PDB format before being exported into the M.O.E. program. Using the M.O.E. with normal technique to perform docking of ligand-receptor bindings, the docked findings were exhibited.

## Conclusion

Using thorough spectroscopic studies, a variety of pyrimidine-based sulfonamide derivatives were successfully developed, synthesized, and structurally verified in this work. The synthetic method made it possible to create a number of heterocyclic frameworks that had a big impact on the molecules’ biological activity. Compounds **18**, **21**, **23**, and **24** exhibited exceptionally strong anticancer activity against HepG2 and MCF-7 cell lines, according to the cytotoxicity screening, with IC_50_ values that are comparable to, and often even closer to, the reference medication 5-fluorouracil. In contrast to 5-FU, which showed noticeably more cytotoxicity against normal cells, these compounds showed modest toxicity toward normal WI-38 and VERO cells, indicating an improved selectivity profile. According to SAR analysis, balanced lipophilicity, optimal heterocyclic fusion, and electron-donating functions are crucial for improving both potency and selectivity. These results were corroborated by molecular docking studies, which demonstrated that the most active compounds, namely compound **23**, had substantial binding affinities to thymidylate synthase due to important hydrogen-bond interactions inside the active site of the enzyme. Compounds **18**, **21**, **23**, and **24** appear to be attractive lead scaffolds for upcoming anticancer drug development, according to the biological evaluation, SAR interpretation, and in-silico docking study taken together. To bring them closer to potential therapeutic uses, further optimization, mechanistic research, and in vivo testing are necessary, given their high potency, favorable safety margins, and robust molecular interactions.

## Supplementary Information

Below is the link to the electronic supplementary material.

Supplementary Material 1

## Data Availability

Data generated or analyzed during this study are included in this published article and submitted as a supplementary file [supplementary data].
