# Supplementary material for: Design, synthesis, molecular docking and cytotoxic evaluation of novel pyrimidine-based sulfonamide derivatives as potent anticancer agents: SAR insights and biological profiling
Source: Sci Rep. 2026 Mar 24;16:9820. doi: 10.1038/s41598-026-41711-z (PMC13018296; doi:10.1038/s41598-026-41711-z)
Supplement: Supplementary file 1 — Supplementary Material 1 [file 41598_2026_41711_MOESM1_ESM.docx]

**Experiment**

**Synthesis of 3-amino-3-(hydroxyimino)-N-(4-(N-(pyrimidin-2-yl)sulfamoyl)phenyl)propenamide (2)**

Compound **1** (0.30 g, 0.001 mol) and hydroxylamine hydrochloride (0.08 g, 0.0012 mol) was refluxed in boiling EtOH (25 mL) with few drops of TEA (3 drops) for 3 h. The obtained solid material on cooling was filtered and recrystallized from ethanol to give compound **2**.

Yield, 73%; m.p. 187-189 °C; IR (KBr): ν_max_, cm^-1^: 3510 (OH), 3430 (NH_2_), 3230, 3210 (2NH), 1675 (amidic CO), 1620 (C=N), 1600 (C=C), 1330 (SO_2_); ^1^H-NMR (DMSO-*d_6_*) *δ* ppm: 3.09 (s, 2H, CH_2_), 6.20 (s, 2H, NH_2_), 6.99-7.07 (m, 1H, pyrimidine-C_5_H), 7.70 (d, 2H, Ar-H), 7.79 (d, 2H, Ar-H), 8.51 (d, 2H, pyrimidine-C_4_H + C_6_H), 9.06 (s, 1H, OH), 9.93 (s, 1H, NH), 11.24 (s, 1H, NH); ^13^C-NMR (DMSO-*d_6_*) *δ* ppm: 42.9, 116.0, 118.8 (2C), 129.7, 134.9 (2C), 141.6, 156.6, 158.0 (2C), 165.5, 170.0. MS (m/z, %): 350 (M^+^, 66). Anal. Calced for C_13_H_14_N_6_O_4_S (350.35): C, 44.57; H, 4.03; N, 23.99%. Found: C, 44.51; H, 3.97; N, 23.95%.

**Synthesis of 4-(3-amino-5-oxo-4,5-dihydro-1H-pyrazol-1-yl)-N-(pyrimidin-2-yl)benzenesulfonamide (5)**

A mixture of compound **2** (0.35 g, 0.001 mol) and acetic anhydride (25 mL) was heated in water bath for 8 h. The solid material was separated by filtration, crystallization from hot ethanol to give compound **5**.

Yield, 77%; m.p. 215-217 °C; IR (KBr): ν_max_, cm^-1^: 3410 (NH_2_), 3210 (NH), 1680 (amidic CO), 1618 (C=N), 1595 (C=C), 1310 (SO_2_); ^1^H-NMR (DMSO-*d_6_*) *δ* ppm: 3.19 (s, 2H, CH_2_), 6.40 (s, 2H, NH_2_), 7.10–7.20 (m, 1H, pyrimidine–C_5_H), 7.84 (d, 2H, Ar-H), 8.25 (d, 2H, Ar-H), 8.65 (d, 2H, pyrimidine-C_4_H + C_6_H), 11.27 (s, 1H, NH); ^13^C-NMR (DMSO-*d_6_*) *δ* ppm: 72.0, 115.5, 120.6 (2C), 129.0 (2C), 134.7, 142.9, 157.0 (2C), 160.0, 169.4, 172.3. MS (m/z, %): 332 (M^+^, 54). Anal. Calced for C_13_H_12_N_6_O_3_S (332.34): C, 46.98; H, 3.64; N, 25.29%. Found: C, 46.93; H, 3.59; N, 25.20%.

**Synthesis of 2-(4-oxo-4,5-dihydrothiazol-2-yl)-N-(4-(N-(pyrimidin-2-yl)sulfamoyl)phenyl)acetamide (7)**

A mixture of thioglycolic acid (0.09 mL, 0.001 mol) in glacial acetic acid (20 mL), compound **1** (0.30 g, 0.001 mol) was refluxed for 6h. The yield of solid material was obtained in cooling to room temperature was crystallized from hot ethanol to give compound **7**.

Yield, 71%; m.p. 191-193 °C; IR (KBr): ν_max_, cm^-1^: 3290-3250 (2NH), 1680, 1675 (2 amidic CO), 1620 (C=N), 1610 (C=C), 1330 (SO_2_); ^1^H-NMR (DMSO-*d_6_*) *δ* ppm: 3.10 (s, 2H, CH_2_), 4.04 (s, 2H, thiazole-CH_2_), 7.03-7.13 (m, 1H, pyrimidine–C_5_H), 7.80 (d, 2H, Ar-H), 7.90 (d, 2H, Ar-H), 8.54 (d, 2H, pyrimidine-C_4_H + C_6_H), 9.84 (s, 1H, NH), 11.40 (s, 1H, NH); ^13^C-NMR (DMSO-*d_6_*) *δ* ppm: 38.1, 40.8, 114.9, 117.9 (2C), 129.4 (2C), 134.8, 141.1, 157.5 (2C), 163.9 (2C), 169.5, 176.9. MS (m/z, %): 391 (M^+^, 60). Anal. Calced for C_15_H_13_N_5_O_4_S_2_ (391.42): C, 46.03; H, 3.35; N, 17.89%. Found: C, 46.00; H, 3.28; N, 17.82%.

**Synthesis of 2-(4,6-diamino-3,5-dicyanopyridin-2-yl)-N-(4-(N-(pyrimidin-2-yl)sulfamoyl)phenyl)acetamide (8)**

**Method A.**

A mixture of **1** (0.30 g, 0.001 mol) and malononitrile (0.13 g, 0.002 mol) was refluxed in boiling ethanol (25 mL) with few drops of piperidine (0.5 mL) for 3 h. The solid material was filtered and crystallized from ethanol to give **8**.

**Method B.**

A mixture of **1** (0.30 g, 0.001 mol) and malononitrile dimer (0.13 g, 0.001 mol) with few drops of piperidine (0.5 mL) was heated at 140˚C in pressure tube in silicon oil bath for 0.5 h. The separated solid material was washed with ethanol and recrystallization from EtOH to afford **8**.

Yield, 77%; m.p. 215-217 °C; IR (KBr): ν_max_, cm^-1^: 3460 (NH_2_), 3330, 3260 (2NH), 1677 (amidic CO), 1615 (C=N), 1597 (C=C), 1333 (SO_2_); ^1^H-NMR (DMSO-*d_6_*) *δ* ppm: 4.15 (s, 2H, CH_2_), 5.26 (s, 1H, NH), 6.45 (s, 2H, NH_2_), 6.74 (s, 2H, NH_2_), 7.00-7.10 (m, 1H, pyrimidine-C_5_H), 7.74 (d, 2H, Ar–H), 7.99 (d, 2H, Ar-H), 8.40 (d, 2H, pyrimidine-C_4_H + C_6_H), 11.20 (s, 1H, NH); ^13^C-NMR (DMSO-*d_6_*) *δ* ppm: 38.1, 75.2, 86.0, 113.4, 115.0, 117.0, 119.0 (2C), 129.5 (2C), 134.5, 140.9, 157.2 (2C), 163.9 (3C), 168.7, 169.4. MS (m/z, %): 449 (M^+^, 64). Anal. Calced for C_19_H_15_N_9_O_3_S (449.45): C, 50.78; H, 3.36; N, 28.05%. Found: C, 50.72; H, 3.30; N, 28.00%.

**Synthesis of 2-imino-N-(4-(N-(pyrimidin-2-yl)sulfamoyl)phenyl)-2H-chromene-3-carboxamide (9)**

A mixture of compound **1** (0.30 g, 0.001 mol) and salicylaldehyde (0.13 mL, 0.0012 mol) was refluxed in boiling ethanol (25 mL) with few drops of piperidine (0.5 mL) for 3 h. The obtained solid material on cooling was separated and recrystallized from ethanol to give **9**.

Yield, 71%; m.p. 176-178 °C; IR (KBr): ν_max_, cm^-1^: 3210, 3190 (NH), 2219 (CN), 1680 (amidic CO), 1617 (C=N), 1605 (C=C), 1335 (SO_2_); ^1^H-NMR (DMSO-*d_6_*) *δ* ppm: 6.87-7.40 (m, 5H, Ar-H), 7.66 (d, 2H, Ar-H), 7.79 (d, 2H, Ar–H), 8.54 (d, 2H, pyrimidine-C_4_H + C_6_H), 8.71 (s, 1H, chromene-C_4_H), 9.33 (s, 1H, NH), 11.09 (s, 1H, NH), 11.43 (s, 1H, NH); ^13^C-NMR (DMSO-*d_6_*) *δ* ppm: 112.4, 113.9, 116.9 (2C), 117.9 (2C), 122.0, 129.8 (2C), 130.3 (3C), 134.6, 141.8, 154.9, 158.0 (2C), 161.9, 163.8, 170.5. MS (m/z, %): 421 (M^+^, 61). Anal. Calced for C_20_H_15_N_5_O_4_S (421.43): C, 57.00; H, 3.59; N, 16.62%. Found: C, 56.95; H, 3.54; N, 16.57%.

**Synthesis of 2-cyano-2-(3,3-dimethyl-5-oxocyclohexylidene)-N-(4-(N-(pyrimidin-2-yl)sulfamoyl)phenyl)acetamide (10)**

A mixture of cyanoacetanilide **1** (0.30 g, 0.001 mol) and dimedone (0.17 mL, 0.001 mol) was refluxed in boiling EtOH (25 mL) with 4 drops of piperidine for 3 h. The solid material was separated and recrystallization from dry EtOH to afford **10**.

Yield, 78%; m.p. 187-189 °C; IR (KBr): ν_max_, cm^-1^: 3440 (NH_2_), 3320, 3290 (NH), 1685 (amidic CO), 1620 (C=N), 1600 (C=C), 1325 (SO_2_); ^1^H-NMR (DMSO-*d_6_*) *δ* ppm: 1.07 (s, 6H, 2CH_3_), 1.78 (s, 2H, CH_2_), 2.22 (s, 2H, CH_2_), 3.04 (s, 2H, CH_2_), 6.94-7.05 (m, 1H, pyrimidine-C_5_H), 7.40 (d, 2H, Ar-H), 7.74 (d, 2H, Ar–H), 8.45 (d, 2H, pyrimidine-C_4_H + C_6_H), 10.23 (s, 1H, NH), 11.44 (s, 1H, NH); ^13^C-NMR (DMSO-*d_6_*) *δ* ppm: 27.3 (2C), 31.8, 38.3, 45.5, 55.7, 97.5, 114.9, 115.9, 117.6 (2C), 129.5 (2C), 135.6, 140.6, 157.5 (2C), 162.1, 167.8, 169.1, 198.0. MS (m/z, %): 439 (M^+^, 55). Anal. Calced for C_21_H_21_N_5_O_4_S (439.49): C, 57.39; H, 4.82; N, 15.94%. Found: C, 57.33; H, 4.77; N, 15.89%.

**Synthesis of 2-amino-N-(4-(N-(pyrimidin-2-yl)sulfamoyl)phenyl)-4,5,6,7-tetrahydrobenzo[b]thiophene-3-carboxamide (11)**

To a solution of compound **1** (0.30 g, 0.001 mol) in absolute EtOH (20 mL) including catalytically morpholine (0.5 mL), cyclohexanone (0.092 g, 0.001 mol), and elemental sulfur (0.03 g, 0.001 mol) were added. Continuously stirring reaction at 60 ̊C was conducted for 3 h, and then the reaction contents were added drop wise to a crushed ice/water blend acidified by few drops of hydrochloric acid. The yielded precipitate that formed was isolated by filtration, air dried, and recrystallized *via* a mixture of DMF and ethanol (3:1) to afford compound **11**.

Yield, 73%; m.p. 188-190 °C; IR (KBr): ν_max_, cm^-1^: 3450 (NH_2_), 3260 (NH), 3210 (NH), 1675 (CO amide), 1622 (C=N), 1600 (C=C), 1360 (SO_2_); ^1^H-NMR (DMSO-*d_6_*) *δ* ppm: 1.79-1.82 (m, 4H, 2CH_2_), 2.59-2.63 (m, 4H, 2CH_2_), 7.03-7.15 (m, 1H, pyrimidine-C_5_H), 7.48 (s, 2H, NH_2_), 7.75 (d, 2H, Ar-H), 7.99 (d, 2H, Ar-H), 8.51 (d, 2H, pyrimidine-C_4_H + C_6_H), 9.59 (s, 1H, NH), 11.46 (s, 1H, NH); ^13^C-NMR (DMSO-*d_6_*) *δ* ppm: 22.1 (2C), 23.6, 25.5, 115.2, 116.4, 119.0 (2C), 125.6, 128.0 (2C), 135.1, 138.8, 141.0, 157.5 (2C), 162.4, 164.9, 169.6. MS (m/z, %): 429 (M^+^, 67). Anal. Calced for C_19_H_19_N_5_O_3_S_2_ (429.51): C, 53.13; H, 4.46; N, 16.31%. Found: C, 53.08; H, 4.39; N, 16.26%.

***General procedure for coupling reaction of 1 with different primary aromatic amine diazonium salts***

1. Preparation of diazonium salt:

A solution of sodium nitrite (0.05 g, 0.001 mol) in cold water (5 mL) was added drop wise to ice cold solution of the appropriate aromatic amine (0.001 mol) namely 4-aminoantipyrine, 4-aminoazobenzene, 3-amino-4,6-dimethyl-1*H*-pyrazolo[3,4-*b*]pyridine, and 2-amino-1*H*-benzo[d]imidazole, containing adequate amount of hydrochloric acid (1.5 mL) was left to stand in ice cold bath for 1 h.

1. Coupling reaction:

To a cold solution of **1** (0.30 g, 0.001 mol) in pyridine (20 mL) was added to appropriate diazonium chloride solution drop wise over a period 25 min. with continuous stirring. The reaction mixture was left overnight in refrigerator. The separate solid material was filtered and recrystallized from ethanol to give compounds **12a**, **12b**, **13** and **14**, respectively.

**N-(1,5-dimethyl-3-oxo-2-phenyl-2,3-dihydro-1H-pyrazol-4-yl)-2-oxo-2-((4-(N-(pyrimidin-2-yl)sulfamoyl)phenyl)amino)acetohydrazonoyl cyanide (12a)**

Yield, 68%; m.p. 166-168 °C; IR (KBr): ν_max_, cm^-1^: 3330-3255 (NH), 2218 (CN), 1680 and 1675 (2 CO), 1615 (C=N), 1596 (C=C), 1350 (SO_2_); ^1^H-NMR (DMSO-*d_6_*) *δ* ppm: 2.25 (s, 3H, CH_3_), 3.15 (s, 3H, N-CH_3_), 5.33 (s, 1H, NH), 6.95-7.09 (m, 1H, pyrimidine-C_5_H), 7.44-7.69 (m, 5H, Ar-H), 7.78 (d, 2H, Ar-H), 7.98 (d, 2H, Ar-H), 8.39 (d, 2H, pyrimidine-C_4_H + C_6_H), 9.86 (s, 1H, NH), 11.22 (s, 1H, NH). MS (m/z, %): 531 (M^+^, 60). Anal. Calced for C_24_H_21_N_9_O_4_S (531.55): C, 54.23; H, 3.98; N, 23.72%. Found: C, 54.15; H, 3.94; N, 23.66%.

**2-Oxo-N-(4-(phenyldiazenyl)phenyl)-2-((4-(N-(pyrimidin-2-yl)sulfamoyl)phenyl)amino)acetohydrazonoyl cyanide (12b)**

Yield, 71%; m.p. 173-175°C; IR (KBr): ν_max_, cm^-1^: 3345-3260 (NH), 2220 (CN), 1677 (C=O), 1620 (C=N), 1605 (C=N), 1560 (N=N), 1330 (SO₂); ^1^H-NMR (DMSO-*d_6_*) *δ* ppm: 6.99-7.87 (m, 12H, Ar-H), 8.50 (d, 2H, pyrimidine-C_4_H + C_6_H), 8.66 (d, 2H, Ar-H), 9.85 (s, 1H, NH), 11.55 (s, 1H, NH), 11.76 (s, 1H, NH). MS (m/z, %): 525 (M^+^, 50). Anal. Calced for C_25_H_19_N_9_O_3_S (525.55): C, 57.14; H, 3.64; N, 23.99%. Found: C, 57.09; H, 3.60; N, 23.92%.

**N-(4,6-dimethyl-1H-pyrazolo[3,4-b]pyridin-3-yl)-2-oxo-2-((4-(N-(pyrimidin-2-yl)sulfamoyl)phenyl)amino)acetohydrazonoyl cyanide (13)**

Yield, 74%; m.p. 181-183 °C; IR (KBr): ν_max_, cm^-1^: 3370 – 3310 (NH), 2219 (CN), 1680 (C=O), 1625 (C=N), 1600 (C=C), 1360 (SO_2_); ^1^H-NMR (DMSO-*d_6_*) *δ* ppm: 2.56 (s, 3H, CH_3_), 2.77 (s, 3H, CH_3_), 6.81 (s, 1H, pyridine-CH), 7.15-7.22 (m, 1H, pyrimidine-C_5_H), 7.71 (d, 2H, Ar-H), 7.88 (d, 2H, Ar-H), 8.49 (d, 2H, pyrimidine-C_4_H + C_6_H), 9.96 (s, 1H, NH), 10.44 (s, 1H, NH), 11.39 (s, 1H, NH), 12.25 (s, 1H, NH); ^13^C-NMR (DMSO-*d_6_*) *δ* ppm: 19.6, 24.7, 92.0, 105.9, 110.5, 116.2, 117.7 (2C), 122.0, 129.5 (2C), 135.9, 140.7, 145.5, 150.4, 155.0, 157.1 (3C), 159.5, 169.7. MS (m/z, %): 490 (M^+^, 69). Anal. Calced for C_21_H_18_N_10_O_3_S (490.50): C, 51.42; H, 3.70; N, 28.56%. Found: C, 51.35; H, 3.66; N, 28.50%.

**N-(1H-benzo[d]imidazol-2-yl)-2-oxo-2-((4-(N-(pyrimidin-2-yl)sulfamoyl)phenyl)amino)acetohydrazonoyl cyanide (14)**

Yield, 77%; m.p. 157-159 °C; IR (KBr): ν_max_, cm^-1^: 3355 (NH), 2220 (CN), 1675 (C=O), 1621 (C=N), 1605 (C=C), 1353 (SO_2_); ^1^H-NMR (DMSO-*d_6_*) *δ* ppm: 7.08-7.20 (m, 1H, pyrimidine-C_5_H), 7.19 (s, 4H, Ar-H), 7.66 (d, 2H, Ar-H), 7.88 (d, 2H, Ar-H), 8.43 (d, 2H, pyrimidine-C_4_H + C_6_H), 9.77 (s, 1H, NH), 10.69 (s, 1H, NH), 11.38 (s, 1H, NH), 12.44 (s, 1H, NH); ^13^C-NMR (DMSO-*d_6_*) *δ* ppm: 106.4, 110.9, 115.8 (3C), 118.9 (2C), 124.1 (2C), 129.9 (2C), 135.6, 136.6 (2C), 140.9 (2C), 157.6 (2C), 160.0, 169.9. MS (m/z, %): 461 (M^+^, 66). Anal. Calced for C_20_H_15_N_9_O_3_S (461.46): C, 52.06; H, 3.28; N, 27.32%. Found: C, 52.00; H, 3.22; N, 27.27%.

**Synthesis of pyrazolo[5,1-c][1,2,4]triazine and imidazo[2,1-c][1,2,4]triazine derivatives (15 and 16)**

***General procedure***

Compounds **15** and **16** were prepared by heating compounds **13** and **14** in glacial acetic acid for 4 h. The reaction mixture was cooled at room temperature and the separated solid material was filtered off, dried and recrystallized ethanol to give the corresponding **15** and **16** respectively.

**4-Imino-8,10-dimethyl-N-(4-(N-(pyrimidin-2-yl)sulfamoyl)phenyl)-4,6-dihydropyrido[2',3':3,4]pyrazolo[5,1-c][1,2,4]triazine-3-carboxamide (15)**

Yield, 71%; m.p. 197-199 °C; IR (KBr): ν_max_, cm^-1^: 3260 (NH), 1681 (C=O), 1622 (C=N), 1600 (C=C), 1360 (SO_2_); ^1^H-NMR (DMSO-*d_6_*) *δ* ppm: 2.61 (s, 3H, CH_3_), 2.68 (s, 3H, CH_3_), 6.87 (s, 1H, pyridine-CH), 6.89-6.99 (m, 1H, pyrimidine-C_5_H), 7.56 (d, 2H, Ar-H), 7.73 (d, 2H, Ar-H), 8.15 (s, 1H, NH), 8.48 (d, 2H, pyrimidine-C_4_H + C_6_H), 9.34 (s, 1H, NH), 9.99 (s, 1H, NH), 11.36 (s, 1H, NH); ^13^C-NMR (DMSO-*d_6_*) *δ* ppm: 19.7, 24.5, 109.9, 114.8, 119.0 (2C), 120.0, 129.4 (2C), 135.6, 140.4, 145.9, 147.6, 157.0 (4C), 159.9, 161.0, 162.9, 170.0. MS (m/z, %): 490 (M^+^, 54). Anal. Calced for C_21_H_18_N_10_O_3_S (490.50): C, 51.42; H, 3.70; N, 28.56%. Found: C, 51.37; H, 3.66; N, 28.49%.

**4-Imino-N-(4-(N-(pyrimidin-2-yl)sulfamoyl)phenyl)-4,10-dihydrobenzo[4,5]imidazo[2,1-c][1,2,4]triazine-3-carboxamide (16)**

Yield, 73%; m.p. 205-207 °C; IR (KBr): ν_max_, cm^-1^: 3310-3260 (NH), 1685 (C=O), 1621 (C=N), 1605 (C=C), 1365 (SO_2_); ^1^H-NMR (DMSO-*d_6_*) *δ* ppm: 5.20 (s, 1H, NH), 6.80-7.00 (m, 5H, Ar-H), 7.72 (d, 2H, Ar-H), 8.00 (d, 2H, Ar-H), 8.30 (d, 2H, pyrimidine-C_4_H + C_6_H), 9.44 (s, 1H, NH), 9.90 (s, 1H, NH), 11.46 (s, 1H, NH); ^13^C-NMR (DMSO-*d_6_*) *δ* ppm: 109.7, 110.7, 115.0, 118.9 (2C), 119.7 (2C), 128.9, 129.4 (2C), 129.9, 134.6, 142.2, 147.3 (2C), 151.4, 156.4 (2C), 160.5, 169.0. MS (m/z, %): 461 (M^+^, 58). Anal. Calced for C_20_H_15_N_9_O_3_S (461.46): C, 52.06; H, 3.28; N, 27.32%. Found: C, 51.99; H, 3.23; N, 27.26%.

**Synthesis of 2-cyano-3-(methylthio)-3-(phenylamino)-N-(4-(N-(pyrimidin-2-yl)sulfamoyl)phenyl)acrylamide (17)**

To a solution of **1** (0.30 g, 0.001 mol) in DMF (25 mL) and KOH (0.056 g, 0.001 mol), phenyl isothiocyanate (0.14 mL, 0.001 mol) was added and the reaction mixture was stirred for 6 h. Dimethyl sulfate (0.11 mL, 0.0012 mol) was added and stirring with continued for 3 h more, then poured into ice cold water. The obtained product was filtered and recrystallizrd from EtOH to give compound **17**.

Yield, 74%; m.p. 184-186 °C; IR (KBr): ν_max_, cm^-1^: 3260 (NH), 2220 (CN), 1677 (C=O), 1622 (C=N), 1603 (C=C), 1355 (SO_2_); ^1^H-NMR (DMSO-*d_6_*) *δ* ppm: 2.30 (s, 3H, CH_3_), 6.65-6.91 (m, 6H, Ar-H), 7.60 (d, 2H, Ar-H), 7.89 (d, 2H, Ar-H), 8.20 (d, 2H, pyrimidine-C_4_H + C_6_H), 10.19 (s, 1H, NH), 10.77 (s, 1H, NH), 11.31 (s, 1H, NH); ^13^C-NMR (DMSO-*d_6_*) *δ* ppm: 16.1, 68.7, 114.2, 115.0, 117.0 (2C), 121.8, 126.0 (2C), 130.4 (4C), 137.6 (2C), 139.7, 157.5 (2C), 164.5, 169.5, 178.8. MS (m/z, %): 466 (M^+^, 62). Anal. Calced for C_21_H_18_N_6_O_3_S_2_ (466.53): C, 54.06; H, 3.89; N, 18.01%. Found: C, 54.00; H, 3.84; N, 17.95%.

**Synthesis of 7-amino-5-(phenylamino)-N-(4-(N-(pyrimidin-2-yl)sulfamoyl)phenyl)-[1,2,4]triazolo[1,5-a]pyrimidine-6-carboxamide (18)**

An equimolar amount of **17** (0.37 g, 0.0008 mol) and 3-amino-1*H*-1,2,4-triazole (0.07 g, 0.0008 mol) was dissolved in pyridine (25 mL) and refluxed for 3 h then left to cool. The reaction mixture was cooled in ice cold water and acidified by few drops of HCl. The isolated solid material was recrystallizrd from EtOH to afford **18**.

Yield, 70%; m.p. 189-191 °C; IR (KBr): ν_max_, cm^-1^: 3440 (NH_2_), 3310–3260 (NH), 1681 (C=O), 1622 (C=N), 1605 (C=C), 1350 (SO_2_); ^1^H-NMR (DMSO-*d_6_*) *δ* ppm: 7.03-7.75 (m, 10H, Ar-H + NH_2_), 7.98 (d, 2H, Ar-H), 8.65 (d, 2H, d, 2H, pyrimidine-C_4_H + C_6_H) 8.77 (s, 1H, triazole-CH), 8.85 (s, 1H, NH), 10.33 (s, 1H, NH), 11.49 (s, 1H, NH); ^13^C-NMR (DMSO-*d_6_*) *δ* ppm: 99.0, 116.0, 117.7 (2C), 118.0 (2C), 123.7, 128.7 (2C), 129.1 (2C), 136.9, 140.0, 142.5, 156.0, 157.4, 159.0 (2C), 166.0, 170.0, 170.6, 171.9. MS (m/z, %): 502 (M^+^, 55). Anal. Calced for C_22_H_18_N_10_O_3_S (502.51): C, 52.58; H, 3.61; N, 27.87%. Found: C, 52.51; H, 3.55; N, 27.82%.

**Synthesis of 2-cyano-2-(imidazolidin-2-ylidene)-N-(4-(N-(pyrimidin-2-yl)sulfamoyl)phenyl)acetamide (19)**

Equimolar mixture of compound **17** (0.37 g, 0.0008 mol) and ethylenediamine (0.05 g, 0.0008 mol) in absolute ethanolic (25 mL) solution, was heated for 3 h, then left to cool. The yielded precipitate was isolated by filtration, and recrystallized from EtOH to afford **19**.

Yield, 68%; m.p. 167-169 °C; IR (KBr): ν_max_, cm^-1^: 3330-3260 (NH), 2220 (CN), 1670 (CO amidic), 1620 (C=N), 1597 (C=C), 1352 (SO_2_); ^1^H-NMR (DMSO-*d_6_*) *δ* ppm: 3.64 (s, 4H, 2CH_2_), 6.35 (s, 2H, 2NH), 7.09-7.19 (m, 1H, pyrimidine-C_5_H), 7.50 (d, 2H, Ar-H), 7.81 (d, 2H, Ar-H), 8.38 (d, 2H, pyrimidine-C_4_H + C_6_H), 10.15 (s, 1H, NH), 11.37 (s, 1H, NH); ^13^C-NMR (DMSO-*d_6_*) *δ* ppm: 42.8 (2C), 56.6, 115.5, 117.8, 118.2 (2C), 129.4 (2C), 135.4, 140.8, 158.0 (2C), 163.0, 169.6, 184.9. MS (m/z, %): 385 (M^+^, 52). Anal. Calced for C_16_H_15_N_7_O_3_S (385.40): C, 49.86; H, 3.92; N, 25.44%. Found: C, 49.79; H, 3.86; N, 25.40%.

**Synthesis of 5-amino-3-(phenylamino)-N-(4-(N-(pyrimidin-2-yl)sulfamoyl)phenyl)-1H-pyrazole-4-carboxamide (20)**

Hydrazine hydrate (0.04 mL, 0.0008 mol) was added to an ethanolic (25 mL) solution of **17** (0.37 g, 0.0008 mol), the mixture was refluxed for 3 h and then allowed to cool. The solid precipitate that yielded was filtered off and recrystallized from EtOH to afford **20**.

Yield, 73%; m.p. 174-176 °C; IR (KBr): ν_max_, cm^-1^: 3460 (NH_2_), 3275 (NH), 1687 (C=O), 1627 (C=N), 1610 (C=C), 1365 (SO_2_); ^1^H-NMR (DMSO-*d_6_*) *δ* ppm: 6.05 (s, 2H, NH_2_), 7.05-7.30 (m, 8H, Ar-H), 7.99 (d, 2H, Ar-H), 8.44 (d, 2H, pyrimidine-C_4_H + C_6_H), 10.25 (s, 1H, NH), 10.60 (s, 1H, NH), 11.35 (s, 1H, NH), 12.69 (s, 1H, NH); ^13^C-NMR (DMSO-*d_6_*) *δ* ppm: 84.2, 114.8, 118.4 (2C), 119.5 (2C), 122.7, 128.1 (4C), 136.8, 141.0, 141.9, 151.7 (2C), 157.8 (2C), 164.0, 169.8. MS (m/z, %): 450 (M^+^, 57). Anal. Calced for C_20_H_18_N_8_O_3_S (450.48): C, 53.33; H, 4.03; N, 24.87%. Found: C, 53.28; H, 3.97; N, 24.81%.

**Synthesis of 5,7-dimethyl-2-(phenylamino)-N-(4-(N-(pyrimidin-2-yl)sulfamoyl)phenyl)pyrazolo[1,5-a]pyrimidine-3-carboxamide (21)**

To a (25 mL) glacial acetic acid solution, equimolar mixture of **20** (0.40 g, 0.0009 mol) and acetylacetone (0.092 mL, 0.0009 mol) was added and heated for 3 h, then left to cool, the reaction contents added drop wise to ice cold water. The formed solid material was isolated by filtration, purified by recrystallization from EtOH/benzene to give **21**.

Yield, 74%; m.p. 178-180 °C; IR (KBr): ν_max_, cm^-1^: 3260 (NH), 1689 (C=O), 1622 (C=N), 1600 (C=C), 1353 (SO_2_); ^1^H-NMR (DMSO-*d_6_*) *δ* ppm: 2.31 (s, 3H, CH_3_), 2.79 (s, 3H, CH_3_), 7.06-7.51 (m, 9H, Ar-H), 8.00 (d, 2H, Ar-H), 8.32 (d, 2H, pyrimidine-C_4_H + C_6_H), 10.29 (s, 1H, NH), 10.69 (s, 1H, NH), 11.31 (s, 1H, NH); ^13^C-NMR (DMSO-*d_6_*) *δ* ppm: 17.7, 24.0, 67.4, 97.3, 108.5, 116.9, 117.9 (2C), 118.0 (2C), 122.6, 129.7 (2C), 129.9 (2C), 135.7, 140.0, 140.7, 144.8, 148.2, 153.7, 157.2 (2C), 166.0, 170.5. MS (m/z, %): 514 (M^+^, 66). Anal. Calced for C_25_H_22_N_8_O_3_S (514.56): C, 58.36; H, 4.31; N, 21.78%. Found: C, 58.30; H, 4.25; N, 21.71%.

**Synthesis of N-(3-(phenylamino)-4-((4-(N-(pyrimidin-2-yl)sulfamoyl)phenyl)carbamoyl)-1H-pyrazol-5-yl)carbonohydrazonoyl dicyanide (23)**

1. Preparation of diazonium salt:

A solution of sodium nitrite (0.062 g, 0.0009 mol, in 2 mL water) was added drop wise to ice cold solution of compound **20** (0.4 g, 0.0009 mol) in a mixture of acetic acid and conc. HCl [(8:2) 10 mL (1/4) Vol.].

1. Coupling reaction:

To a cold solution of malononitrile (0.06 g, 0.0009 mol) in pyridine (10 mL) was added to above formed diazonium chloride solution drop wise over a period 25 min. with continuous stirring. The reaction mixture was left overnight in refrigerator. The separate solid material was filtered and recrystallized from EtOH to give compounds **23**.

Yield, 71%; m.p. 180-182 °C; IR (KBr): ν_max_, cm^-1^: 3340-3313 (NH), 2219, 2198 (2CN), 1702 (C=O), 1622 (C=N), 1605 (C=C), 1573 (N=N), 1350 (SO_2_); ^1^H-NMR (DMSO-*d_6_*) *δ* ppm: 7.15-7.37 (m, 6H, Ar-H), 7.81 (d, 2H, Ar-H), 8.09 (d, 2H, Ar-H), 8.38 (d, 2H, pyrimidine-C_4_H + C_6_H), 10.22 (s, 1H, NH), 10.50 (s, 1H, NH), 10.77 (s, 1H, NH), 11.55 (s, 1H, NH), 12.64 (s, 1H, NH); ^13^C-NMR (DMSO-*d_6_*) *δ* ppm: 84.3 (2C), 111.8 (2C), 115.5, 117.7 (2C), 118.3 (2C), 122.9, 130.3 (4C), 137.0, 139.5, 140.0, 144.9, 152.0, 157.3 (2C), 164.0, 168.8. MS (m/z, %): 527 (M^+^, 62). Anal. Calced for C_23_H_17_N_11_O_3_S (527.52): C, 52.37; H, 3.25; N, 29.21%. Found: C, 52.33; H, 3.20; N, 29.16%.

**Synthesis of 4-amino-3-cyano-7-(phenylamino)-N-(4-(N-(pyrimidin-2-yl)sulfamoyl)phenyl)pyrazolo[5,1-c][1,2,4]triazine-8-carboxamide (24)**

To a (20 mL) glacial acetic acid solution, equimolar mixture of **23** (0.40 g, 0.0008 mol) was added and heated for 3 h, then left to cool, the reaction contents added drop wise to ice cold water. The formed solid material was isolated by filtration, purified by recrystallization from EtOH/DMF to achieve pyrazolo[5,1-*c*][1,2,4]triazine **24**.

Yield, 73%; m.p. 194-196 °C; IR (KBr): ν_max_, cm^-1^: 3460 (NH_2_), 3290 (NH), 2220 (CN), 1690 (C=O), 1622 (C=N), 1600 (C=C), 1350 (SO_2_); ^1^H-NMR (DMSO-*d_6_*) *δ* ppm: 6.39 (s, 2H, NH_2_), 7.09-7.88 (m, 8H, Ar-H), 8.02 (d, 2H, Ar-H), 8.41 (d, 2H, pyrimidine-C_4_H + C_6_H), 10.21 (s, 1H, NH), 10.61 (s, 1H, NH), 11.37 (s, 1H, NH); ^13^C-NMR (DMSO-*d_6_*) *δ* ppm: 55.9, 97.3, 113.5, 115.6, 118.0 (2C), 119.7 (2C), 122.5, 129.6 (4C), 135.9, 140.6, 142.9, 144.4, 148.0, 153.4, 157.0 (2C), 164.9, 169.5. MS (m/z, %): 527 (M^+^, 53). Anal. Calced for C_23_H_17_N_11_O_3_S (527.52): C, 52.37; H, 3.25; N, 29.21%. Found: C, 52.30; H, 3.18; N, 29.15%.


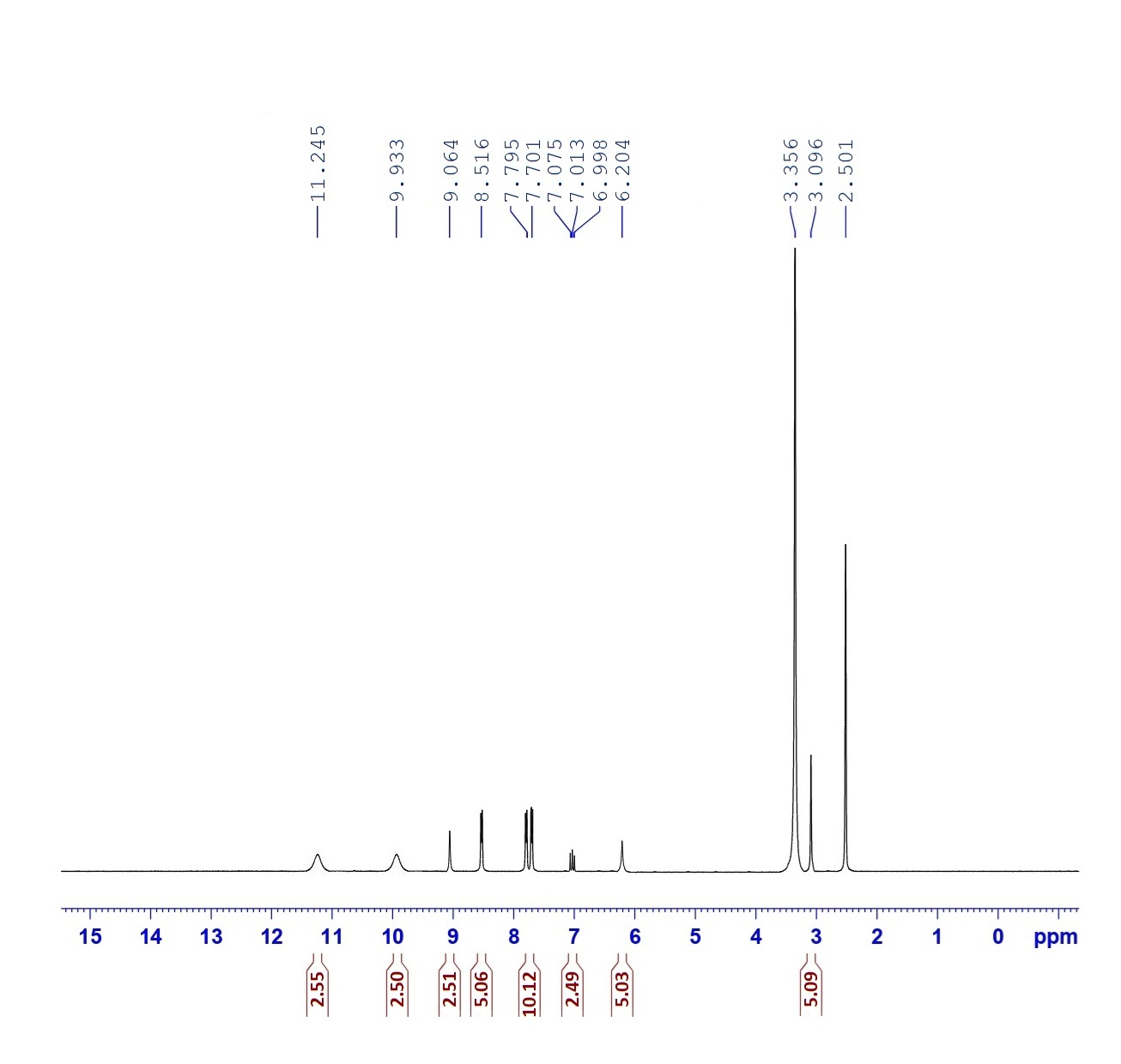


^1^H-NMR of compound **2**


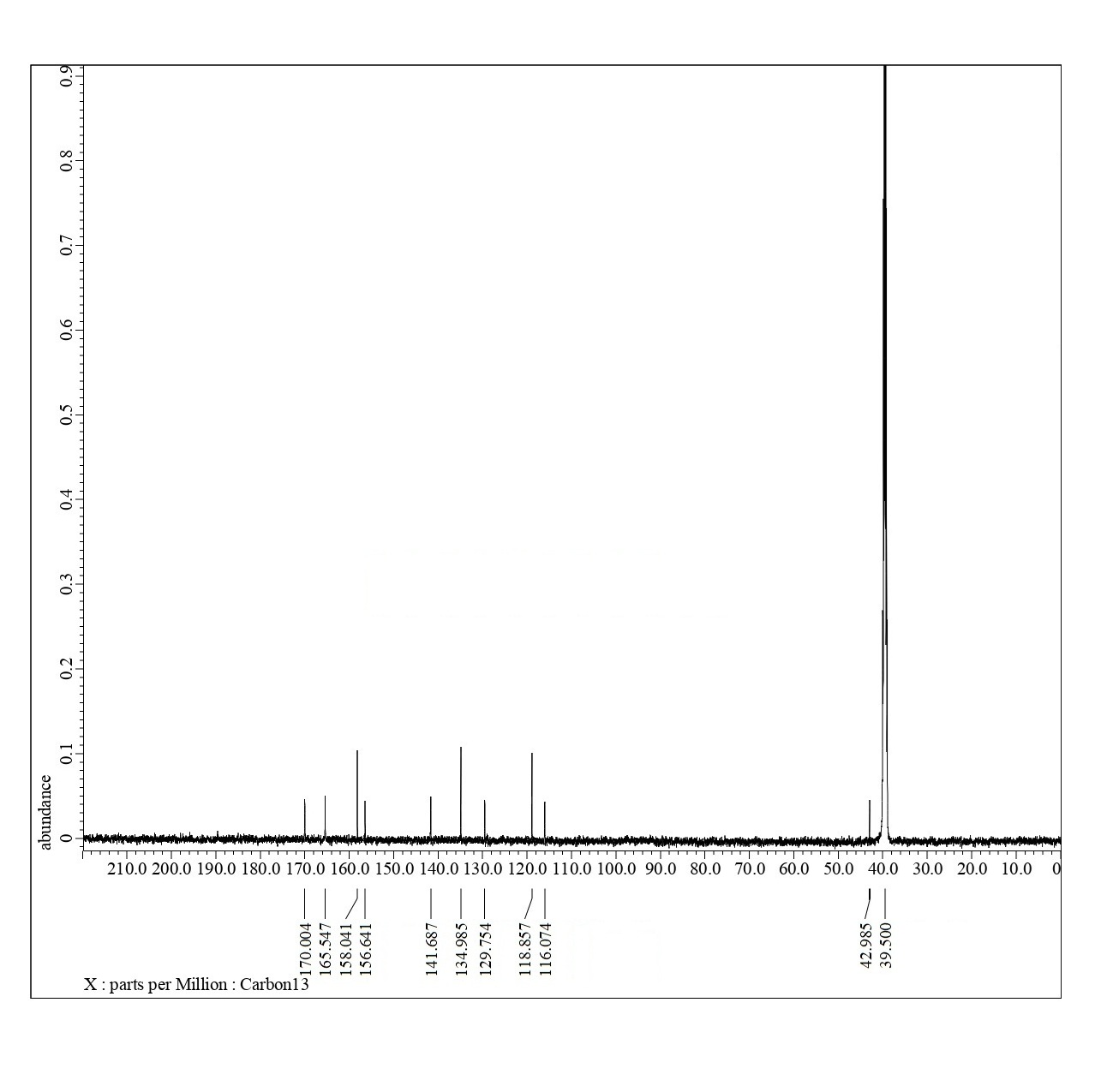


^13^C-NMR of compound **2**

**
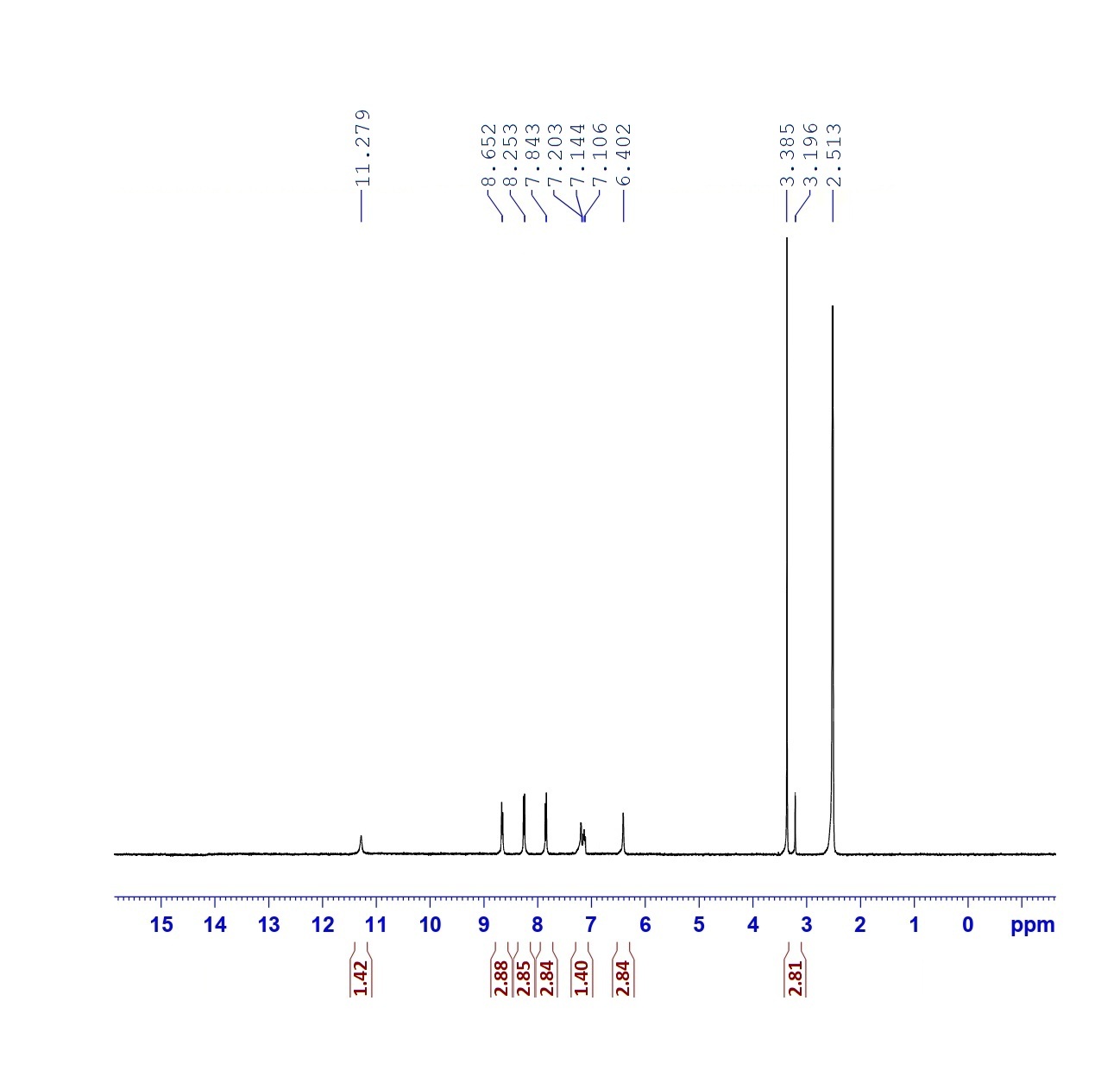
**

^1^H-NMR of compound **5**


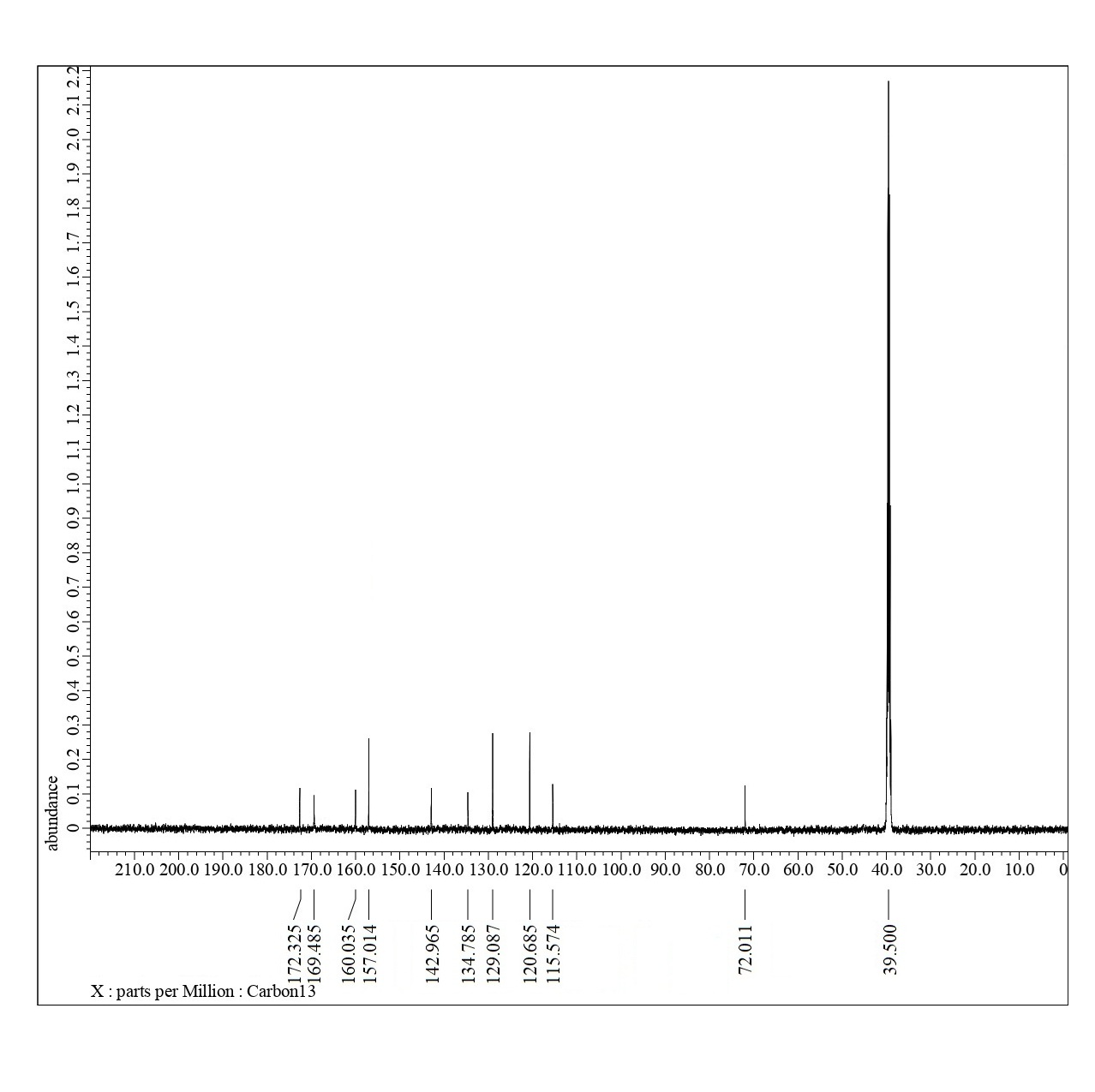


^13^C-NMR of compound **5**

**
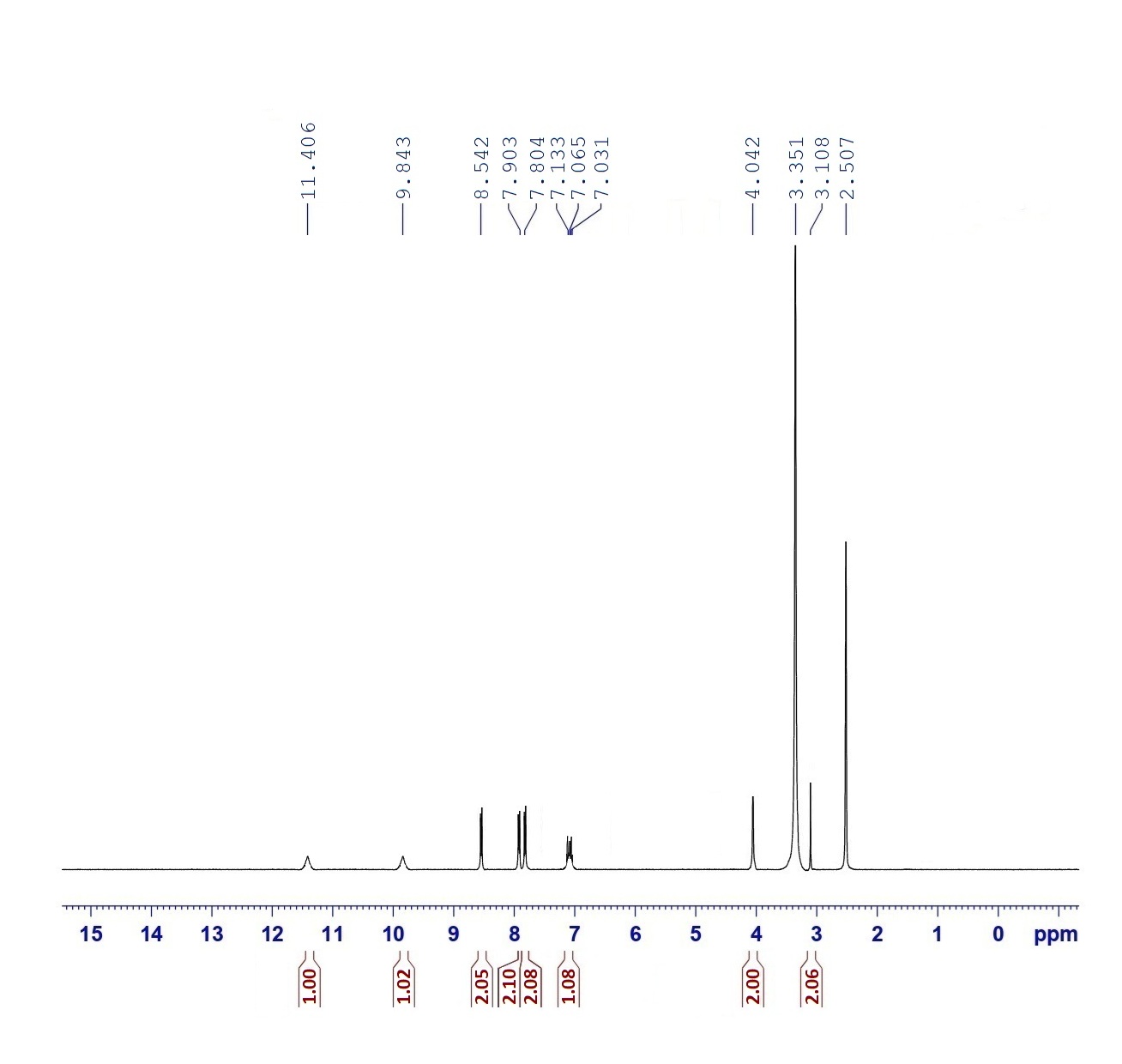
**

^1^H-NMR of compound **7**


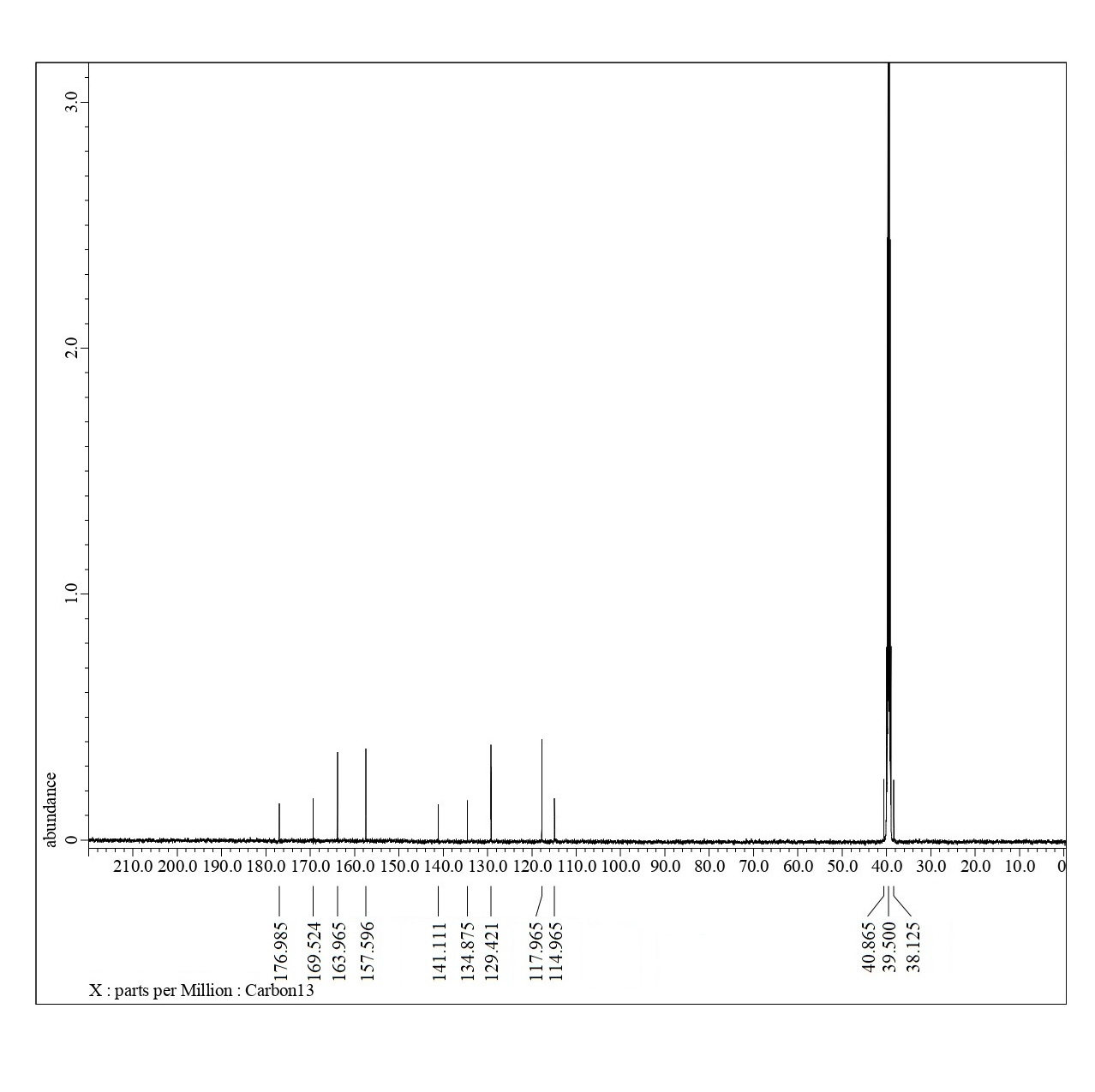


^13^C-NMR of compound **7**

**
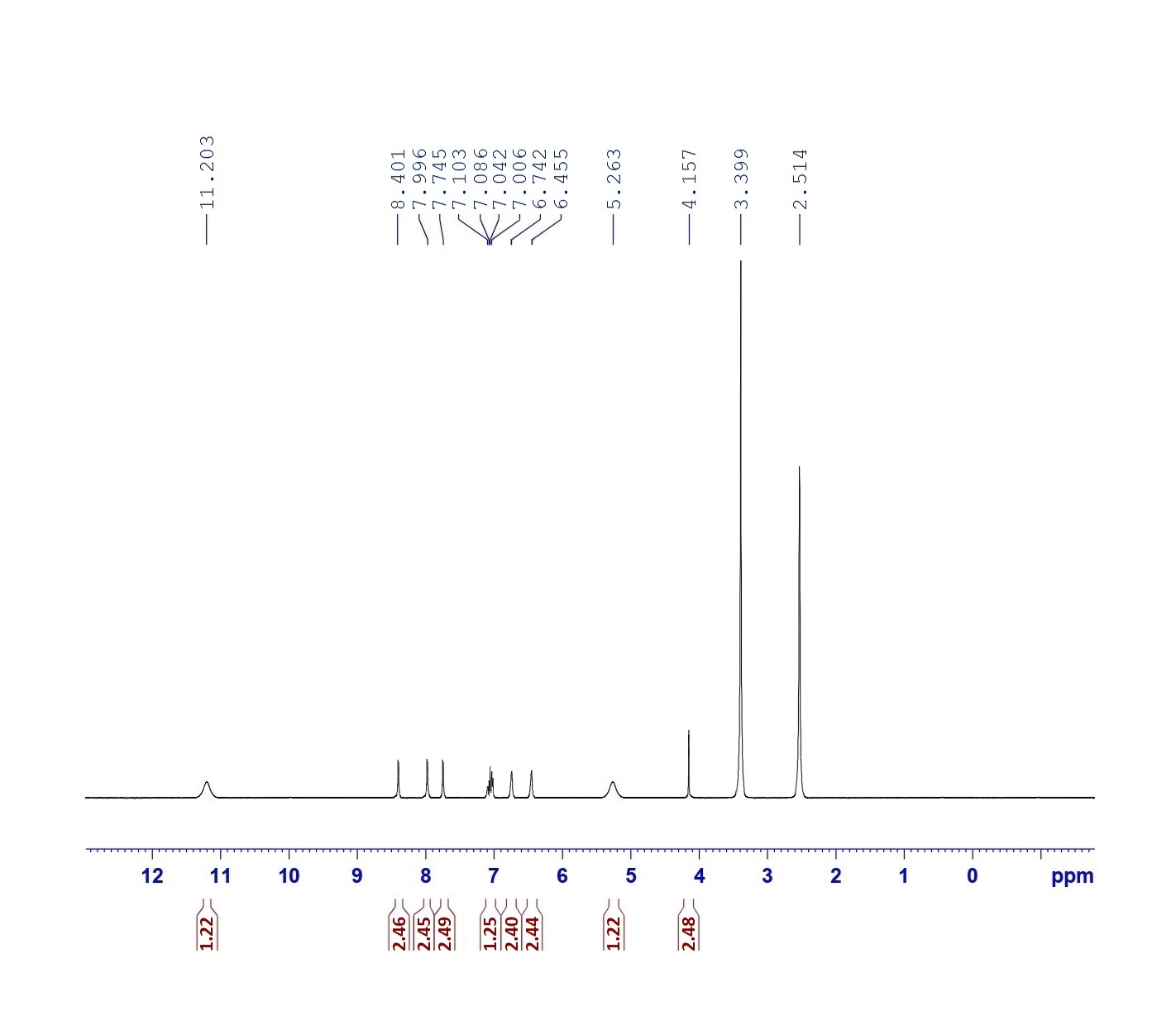
**

^1^H-NMR of compound **8**


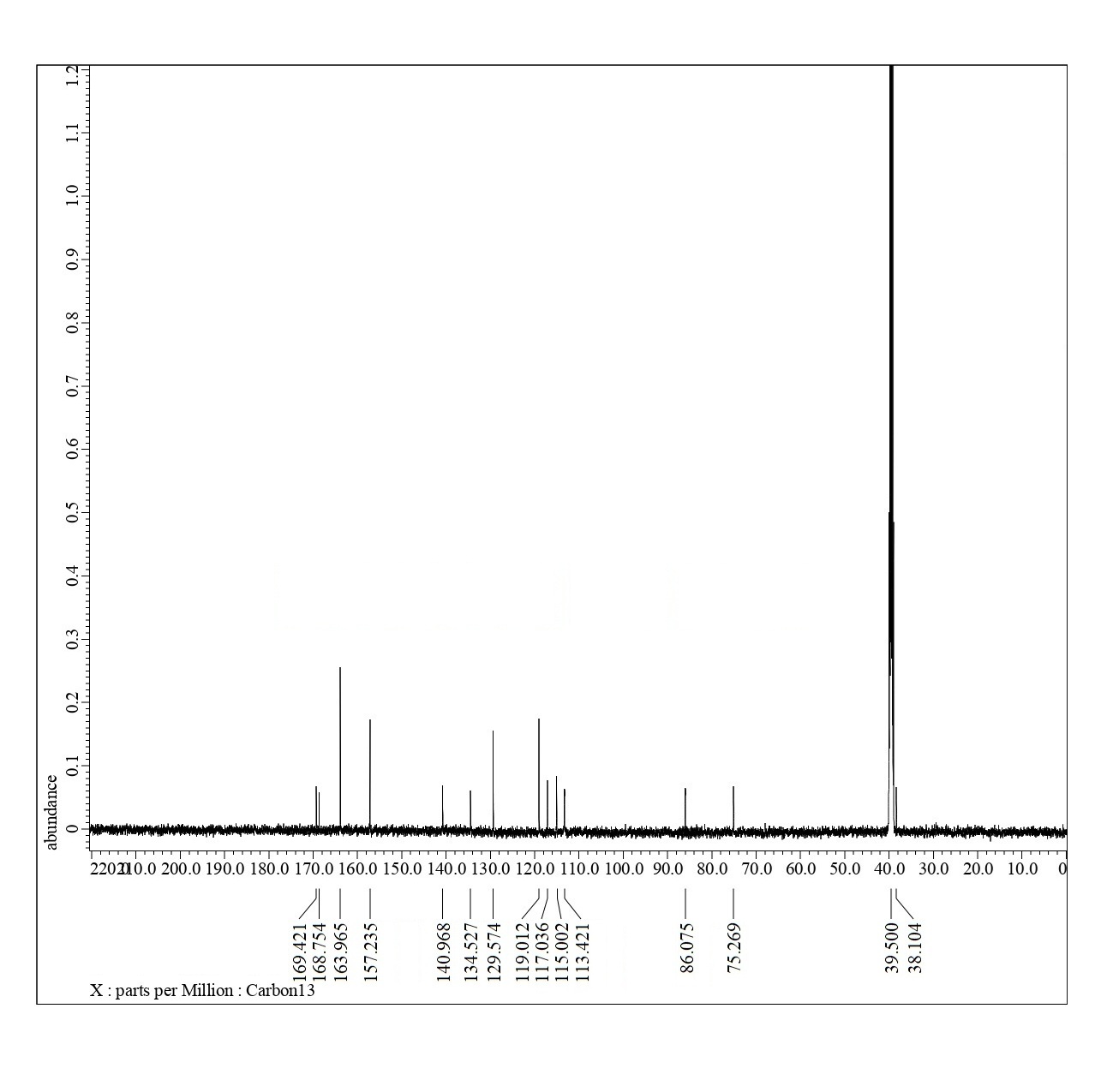


^13^C-NMR of compound **8**


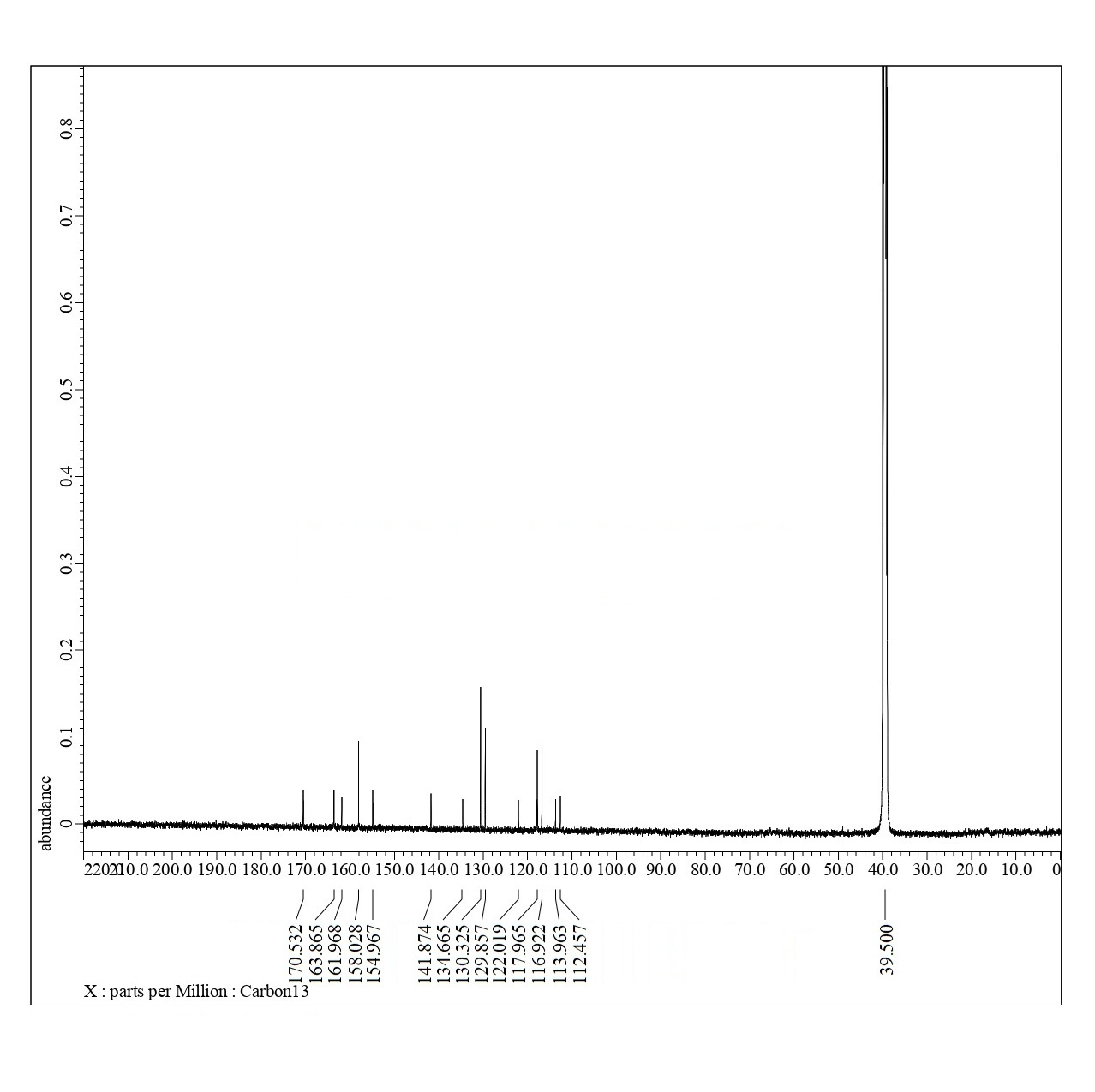


^13^C-NMR of compound **9**

**
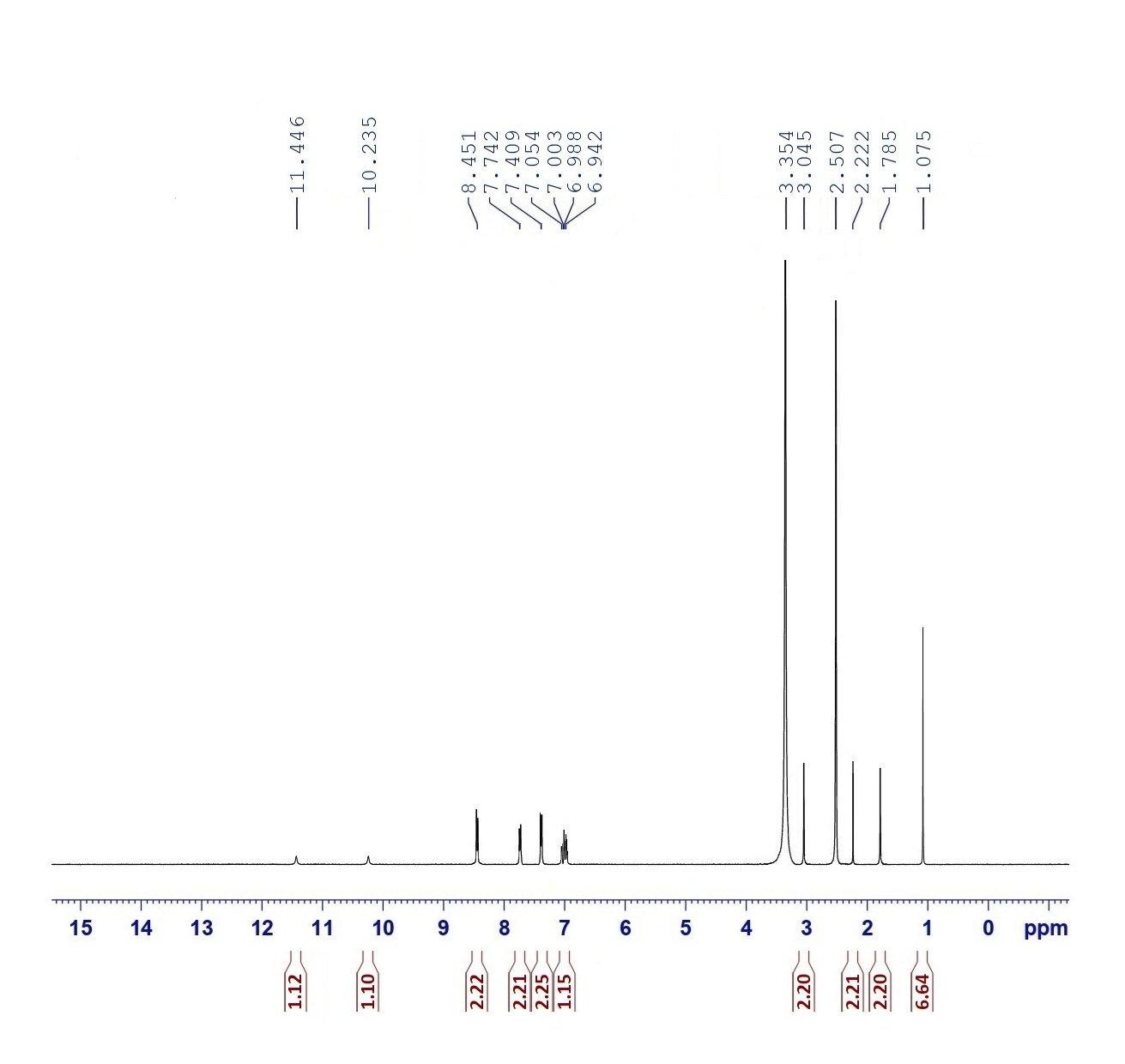
**

^1^H-NMR of compound **10**


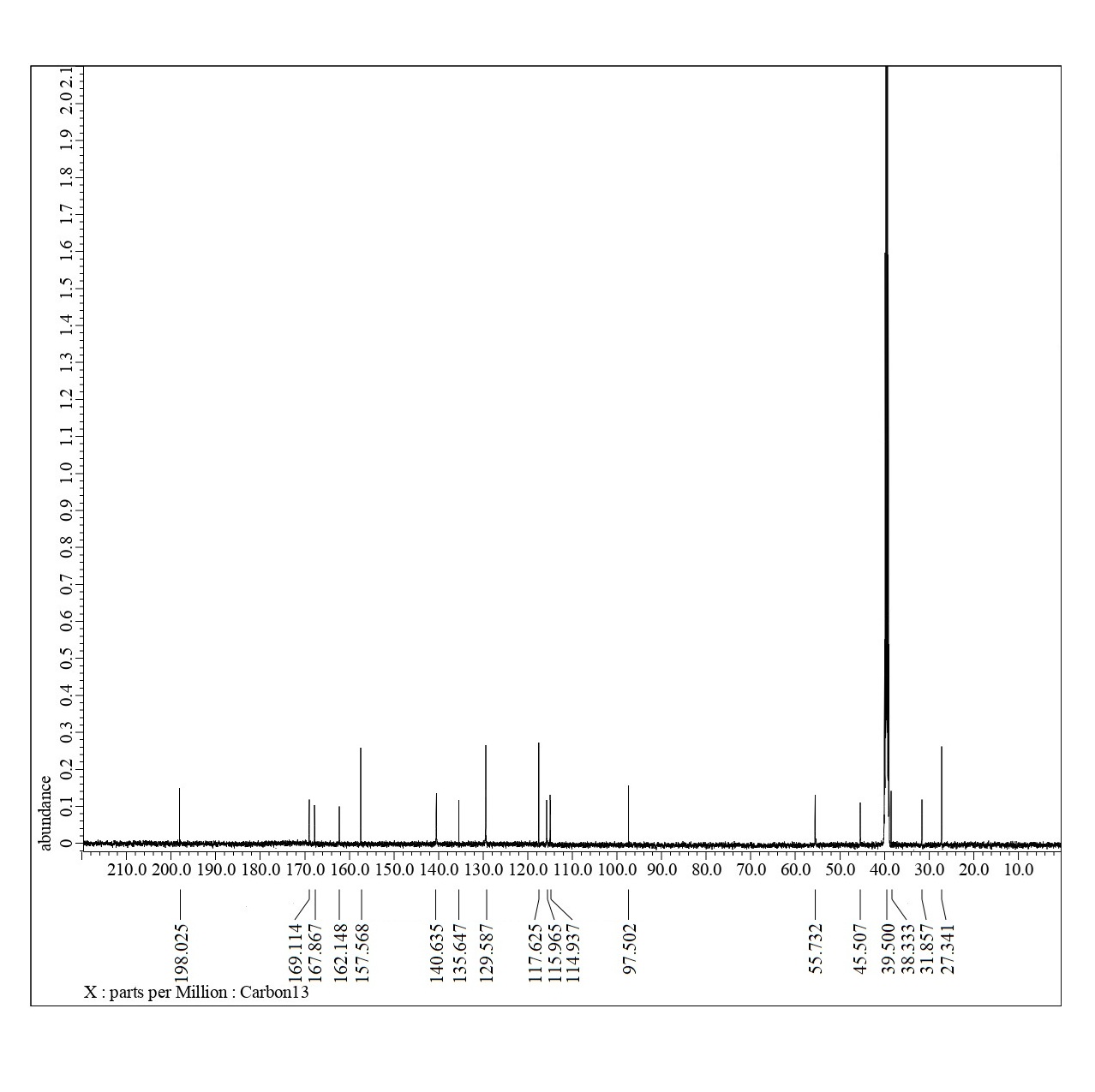


^13^C-NMR of compound **10**


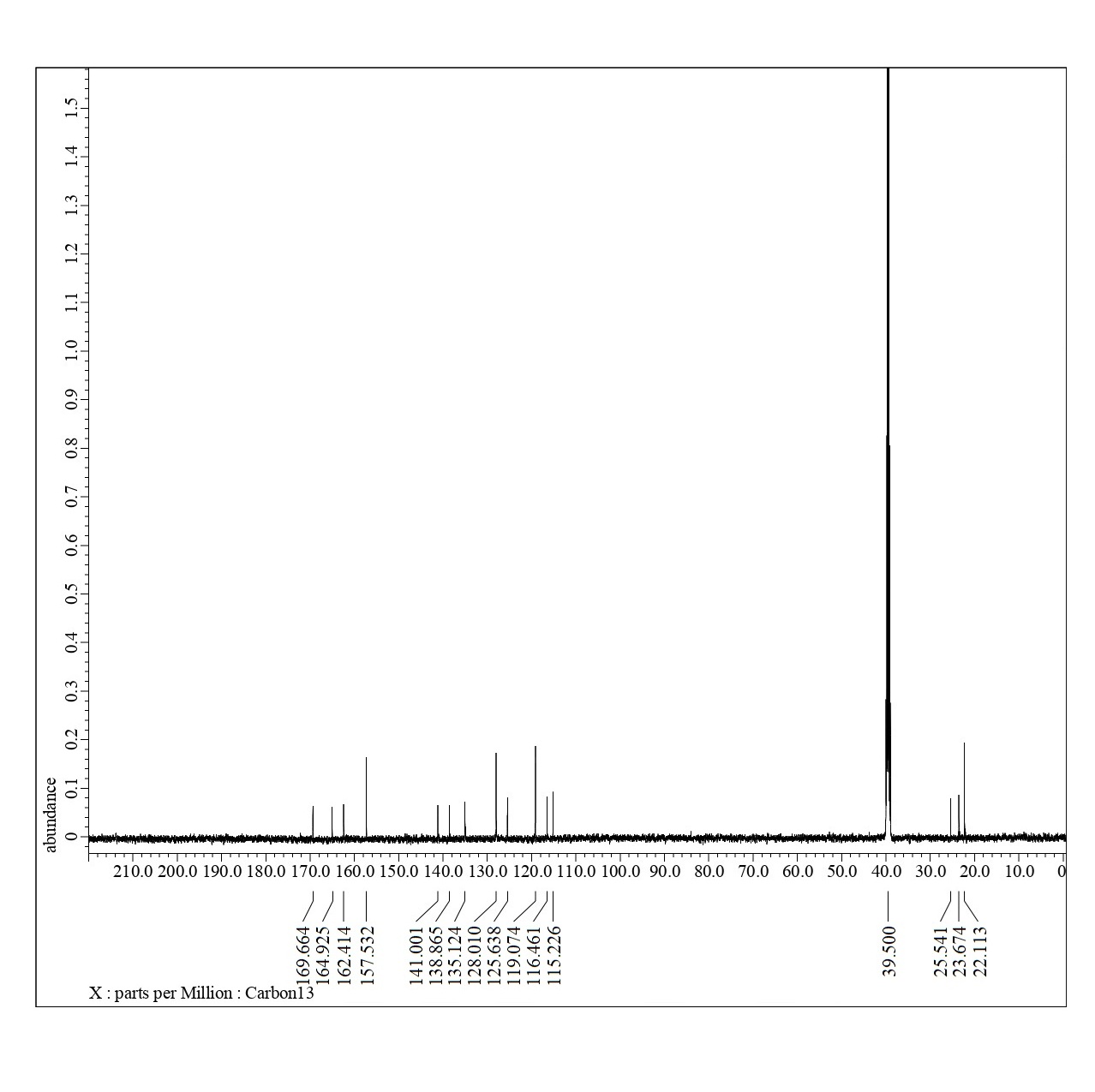


^13^C-NMR of compound **11**


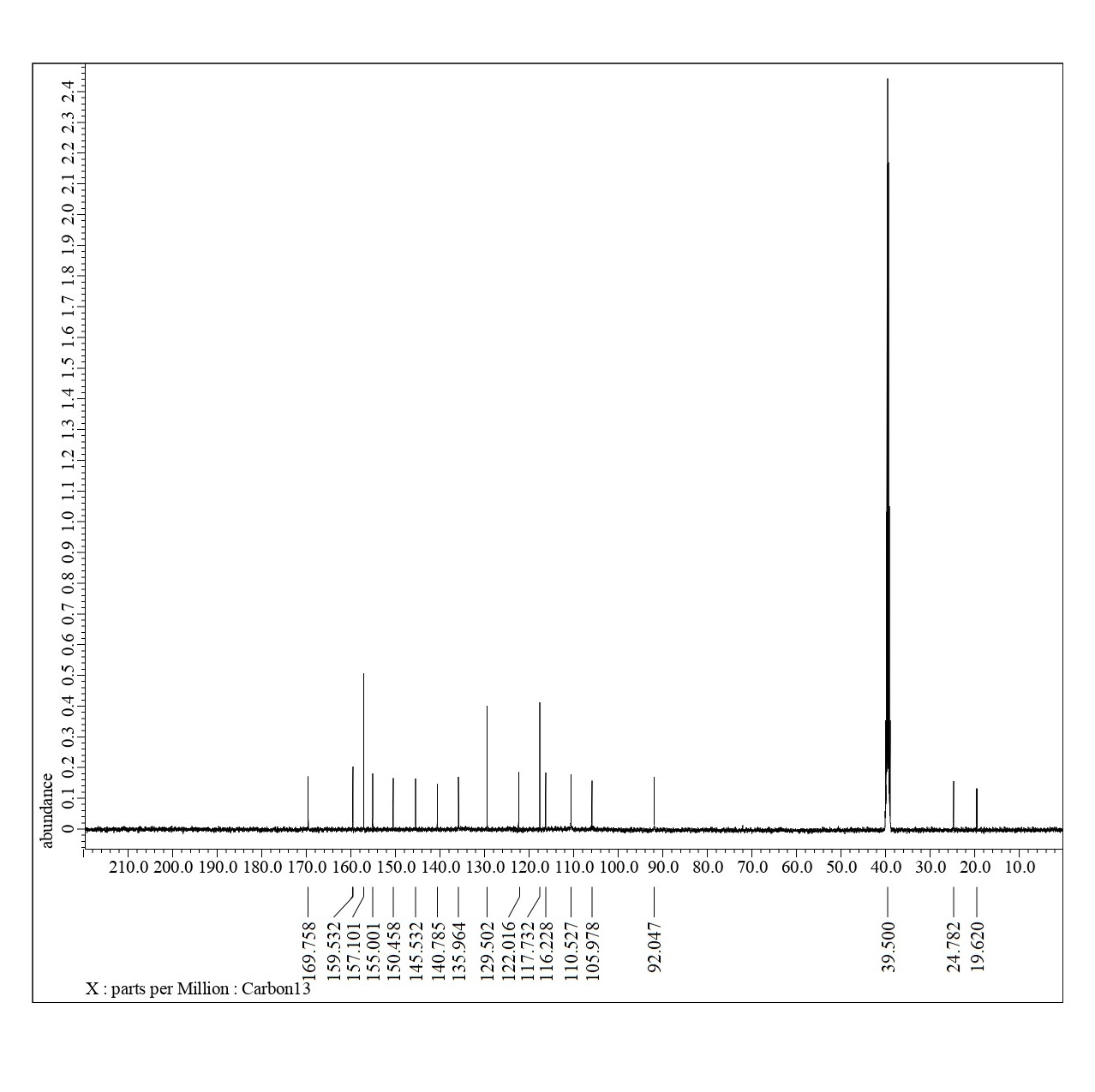


^13^C-NMR of compound **13**


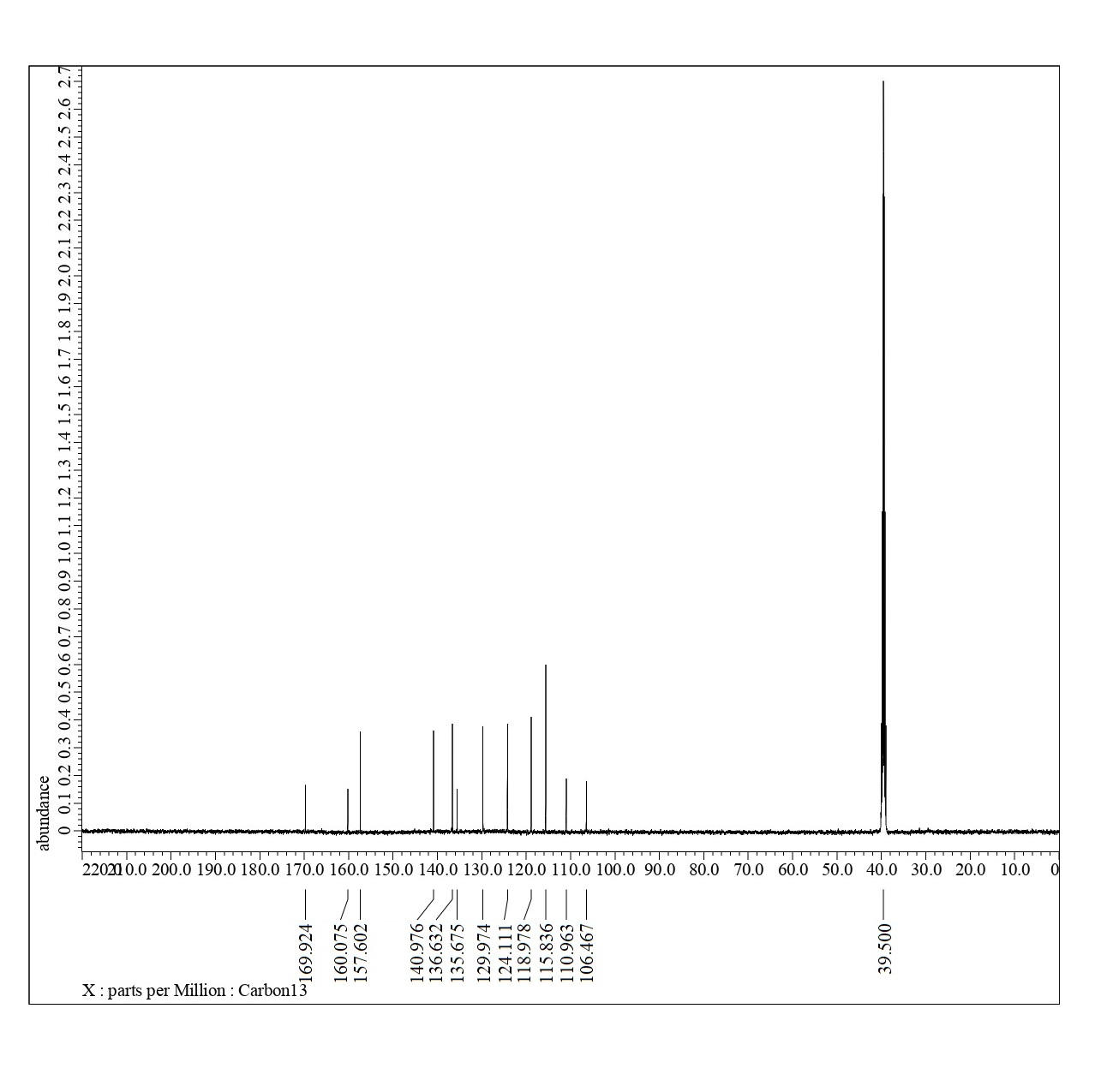


^13^C-NMR of compound **14**


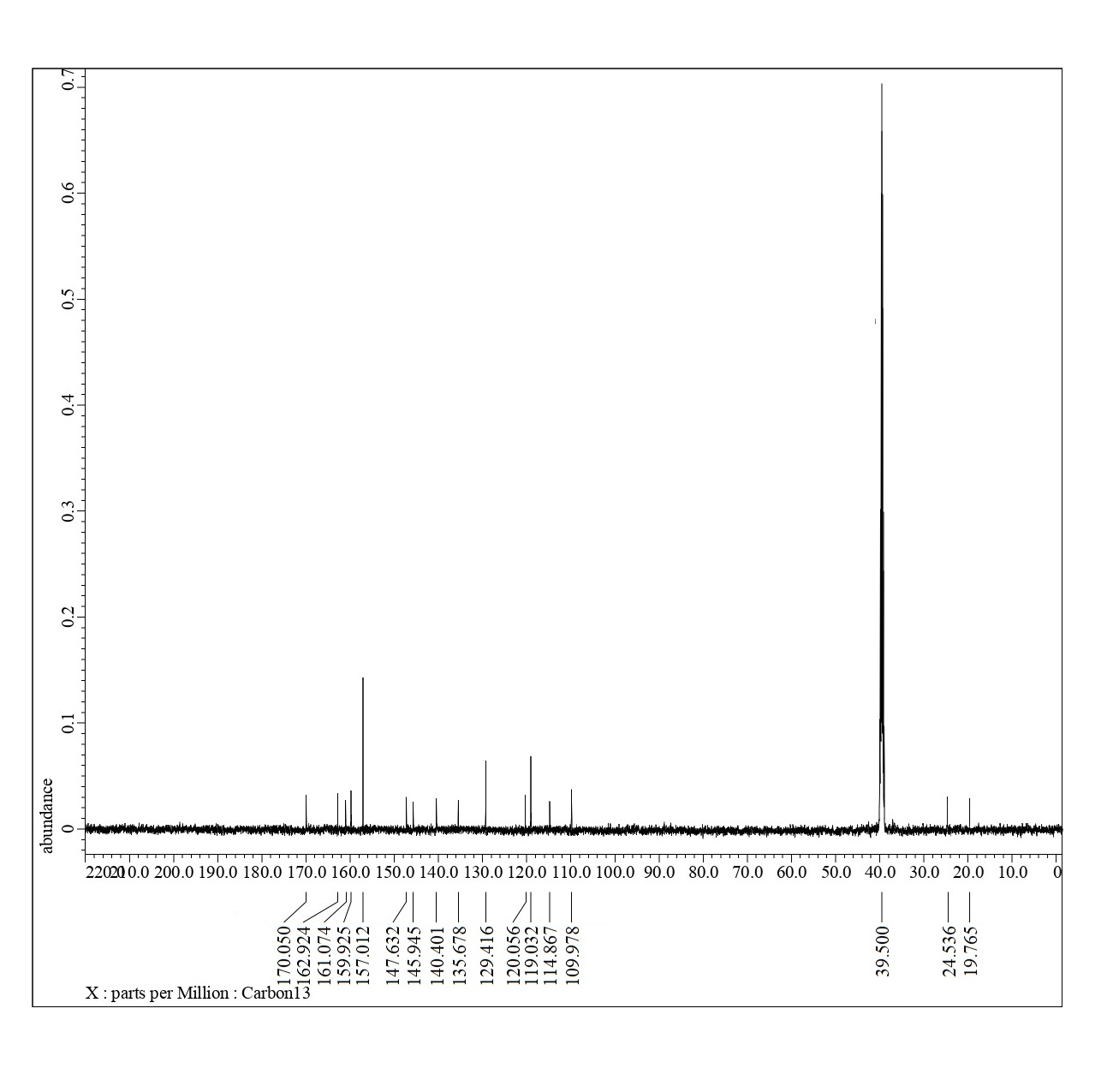


^13^C-NMR of compound **15**

**
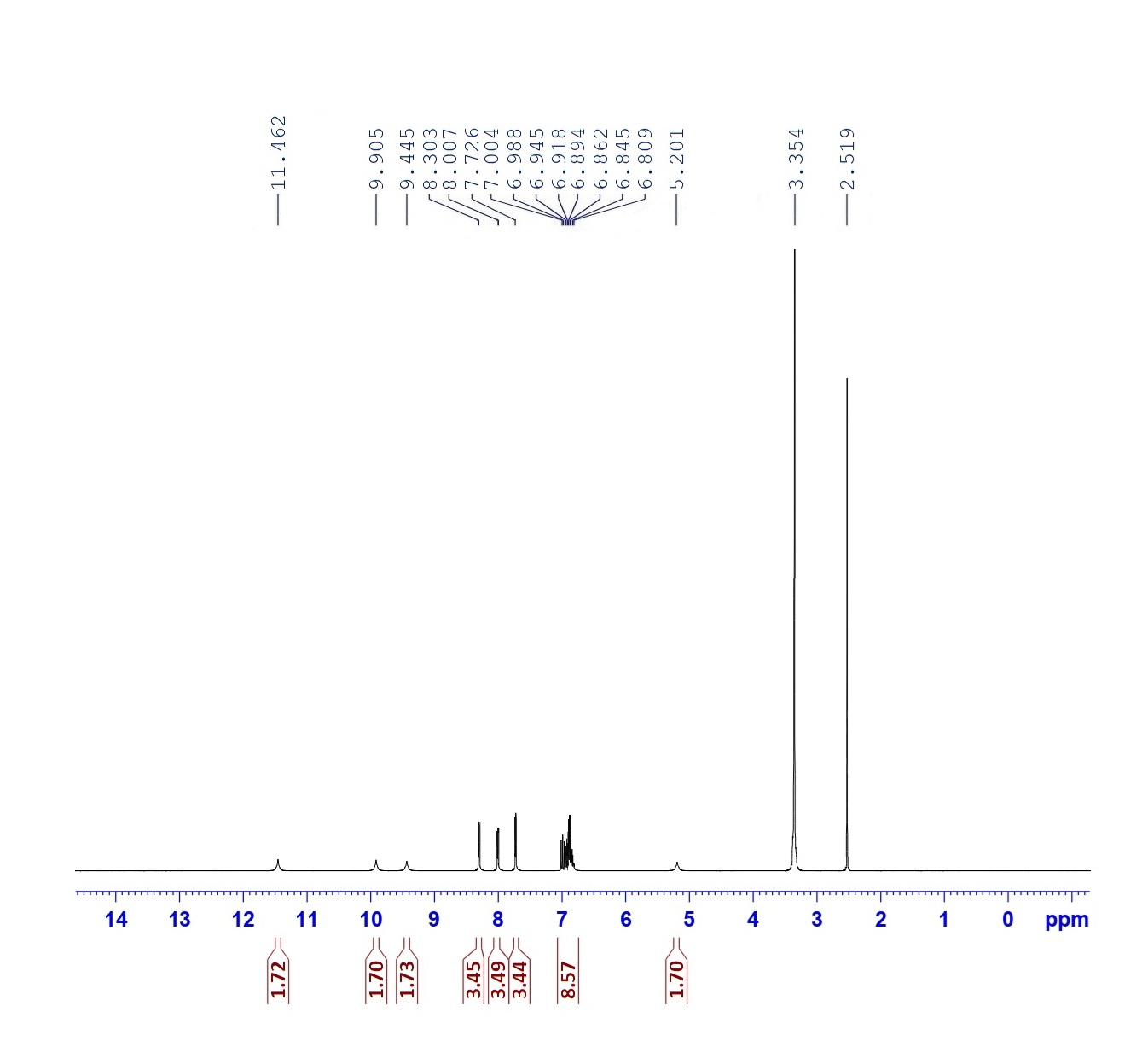
**

^1^H-NMR of compound **16**


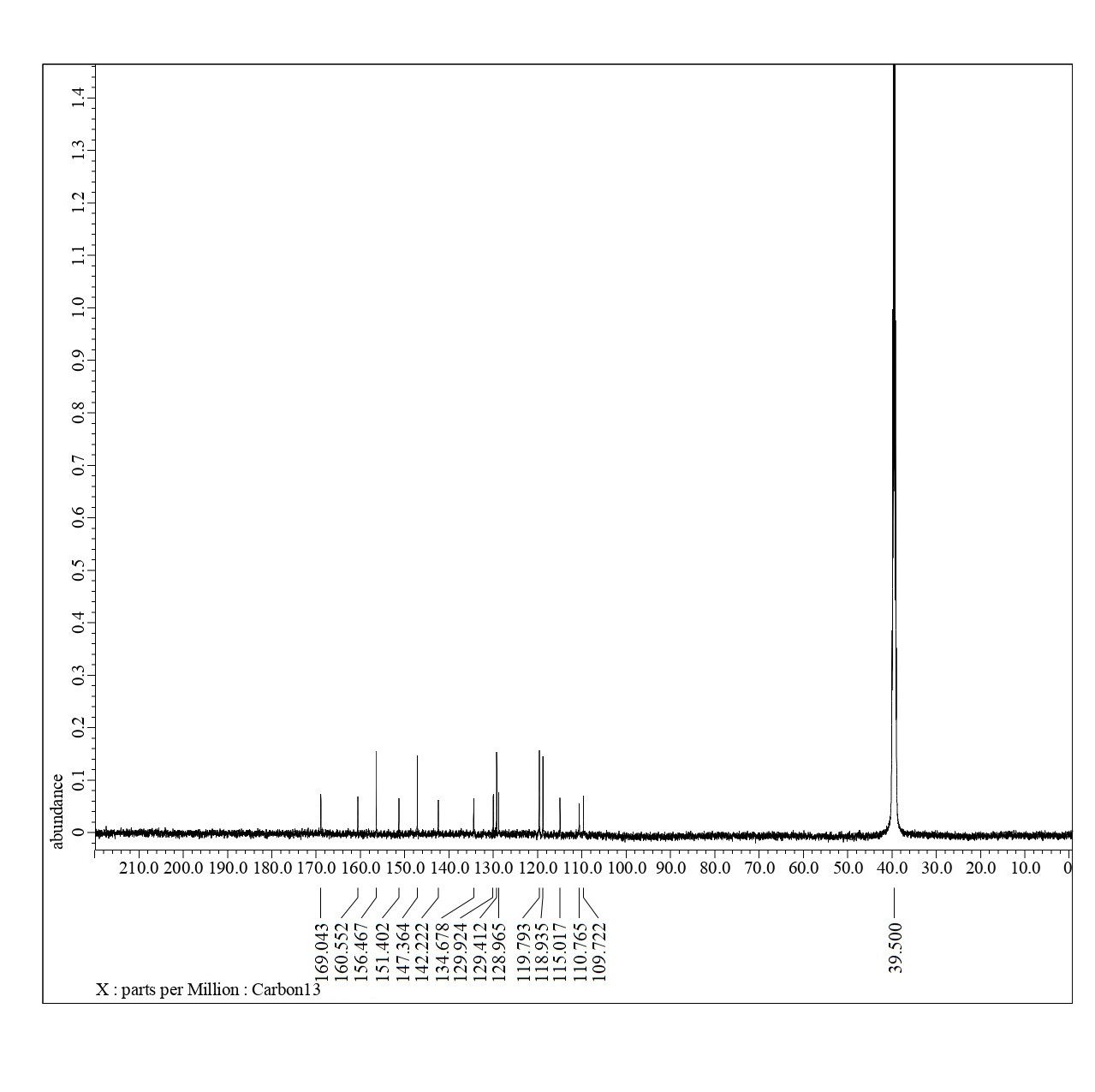


^13^C-NMR of compound **16**

**
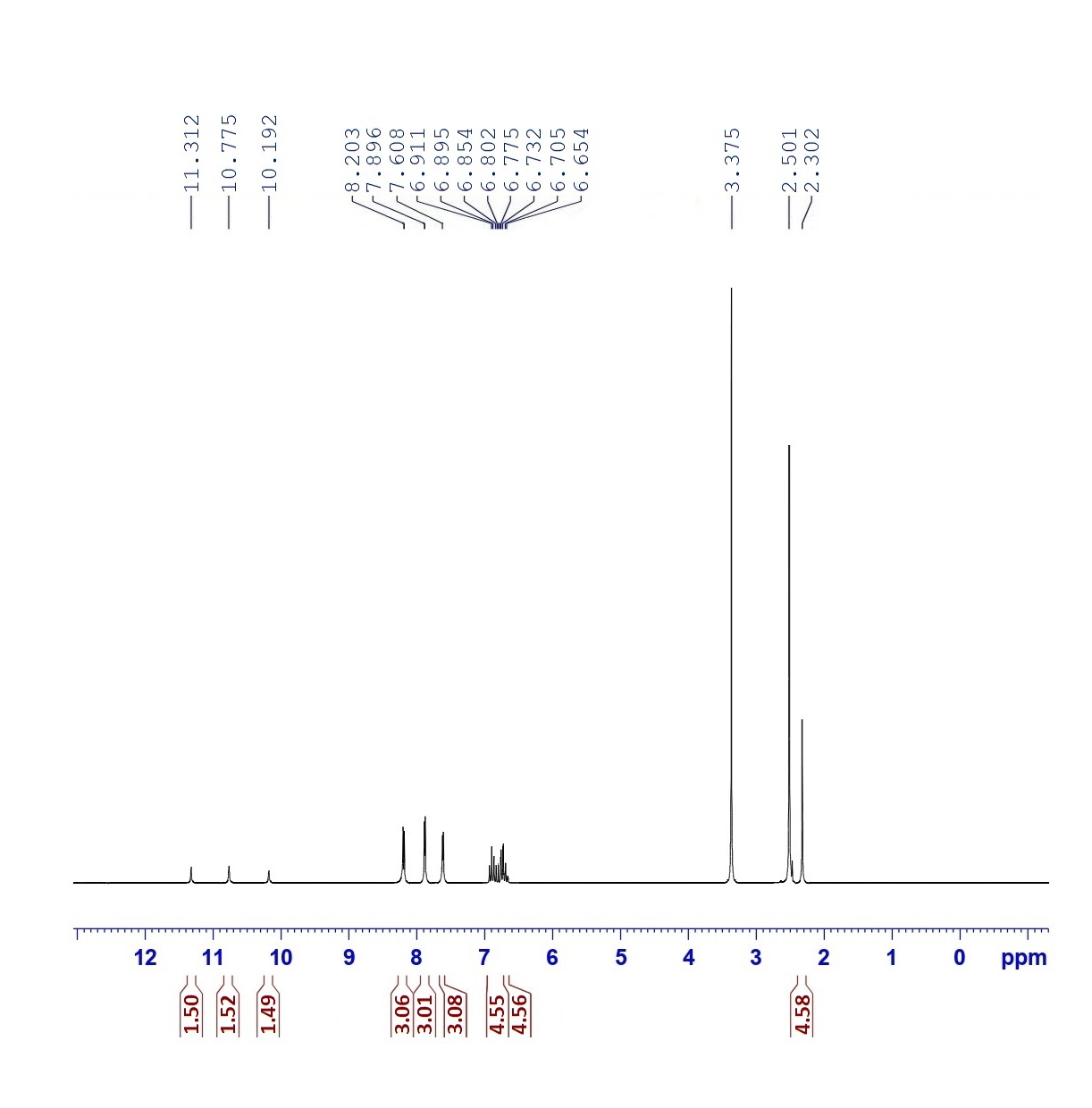
**

^1^H-NMR of compound **17**


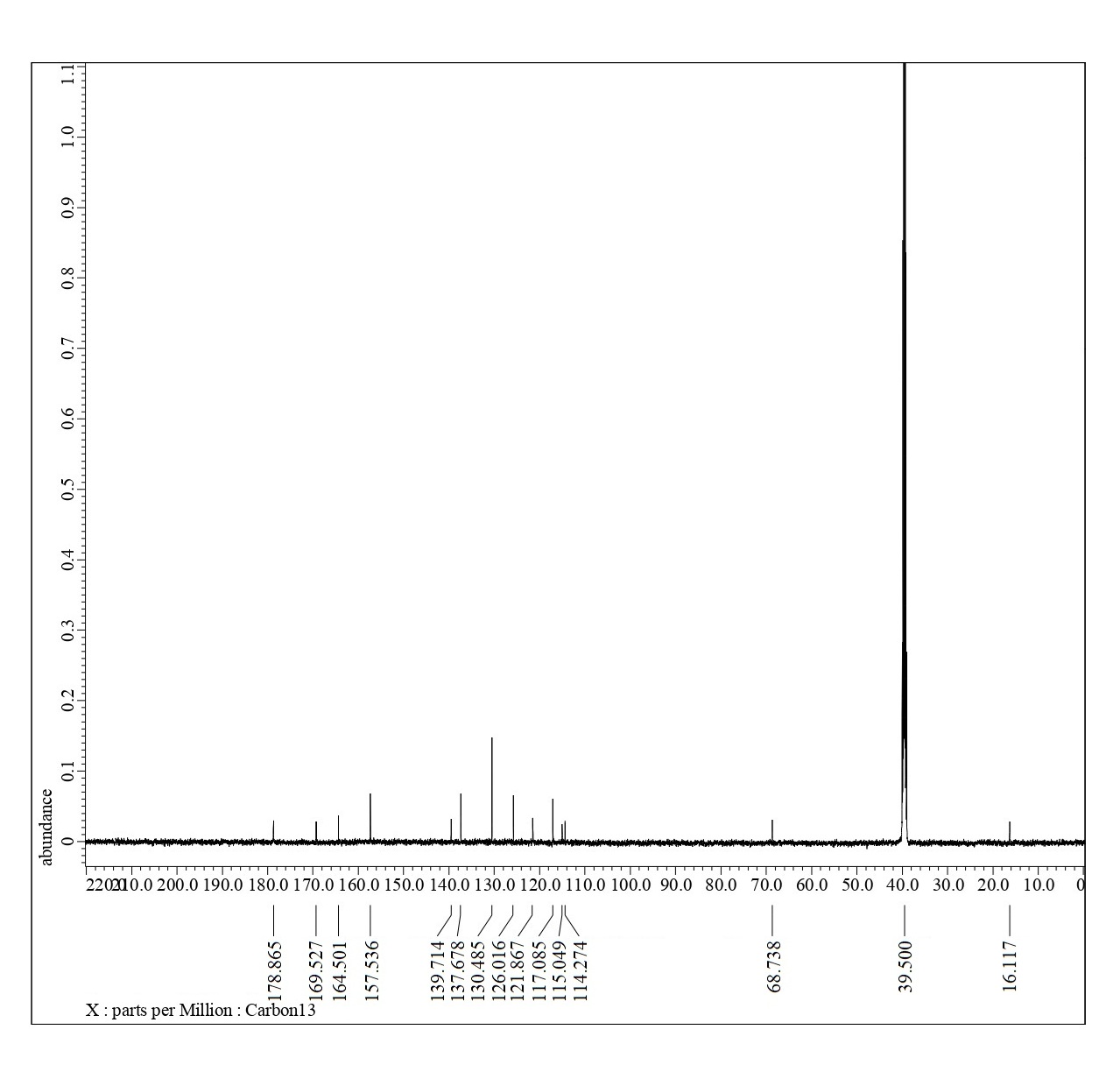


^13^C-NMR of compound **17**


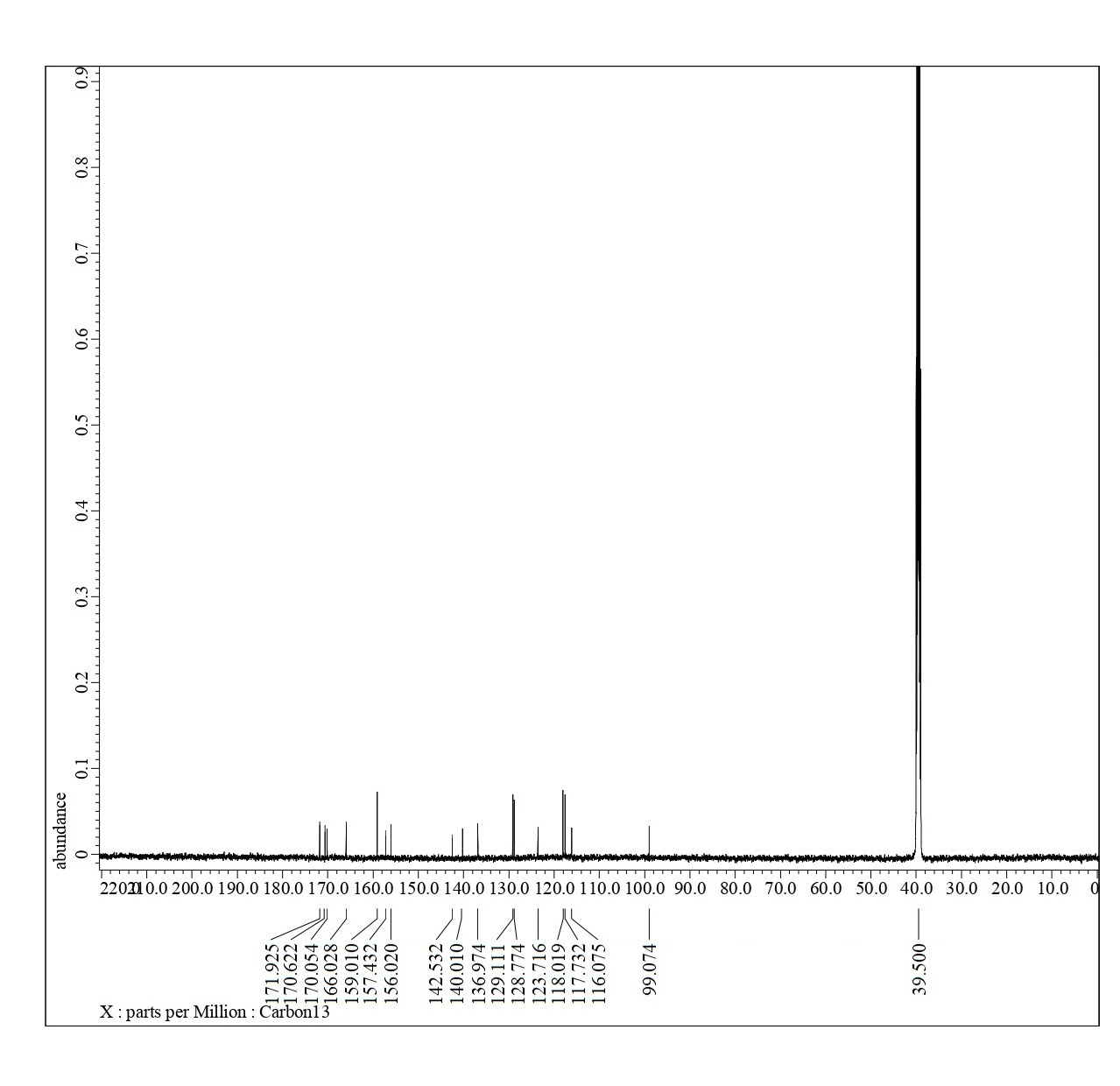


^13^C-NMR of compound **18**

**
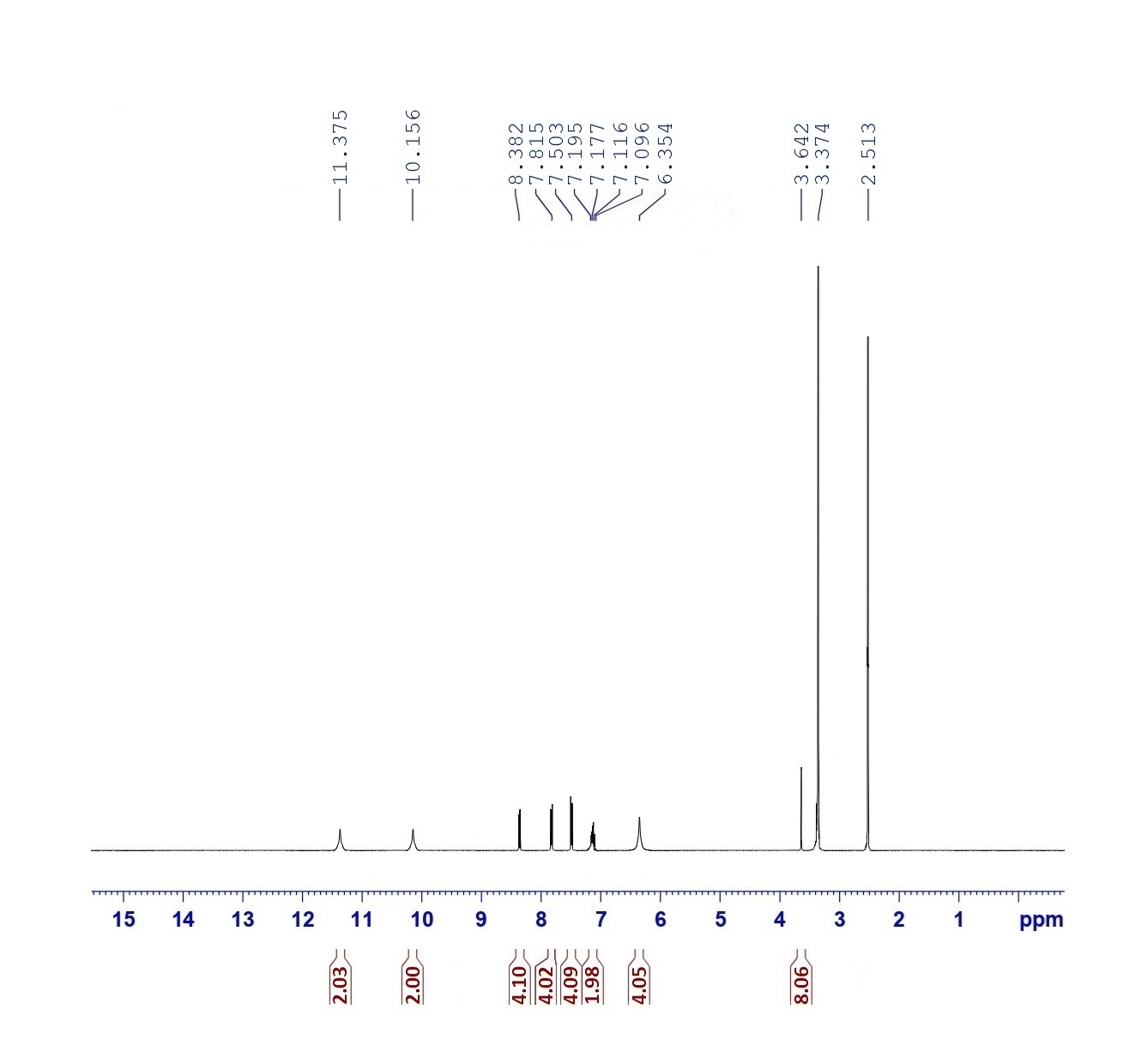
**

^1^H-NMR of compound **19**


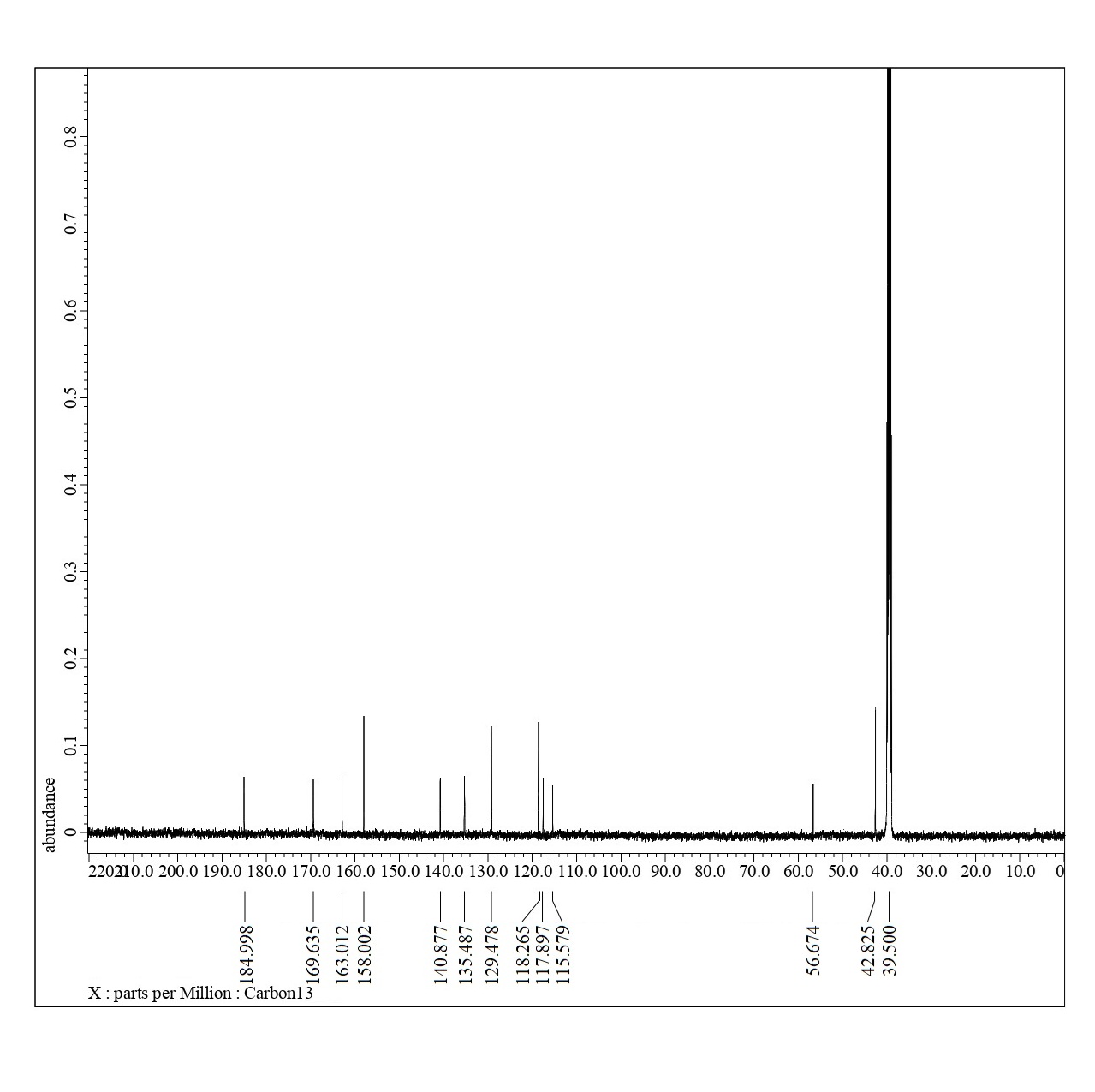


^13^C-NMR of compound **19**

**
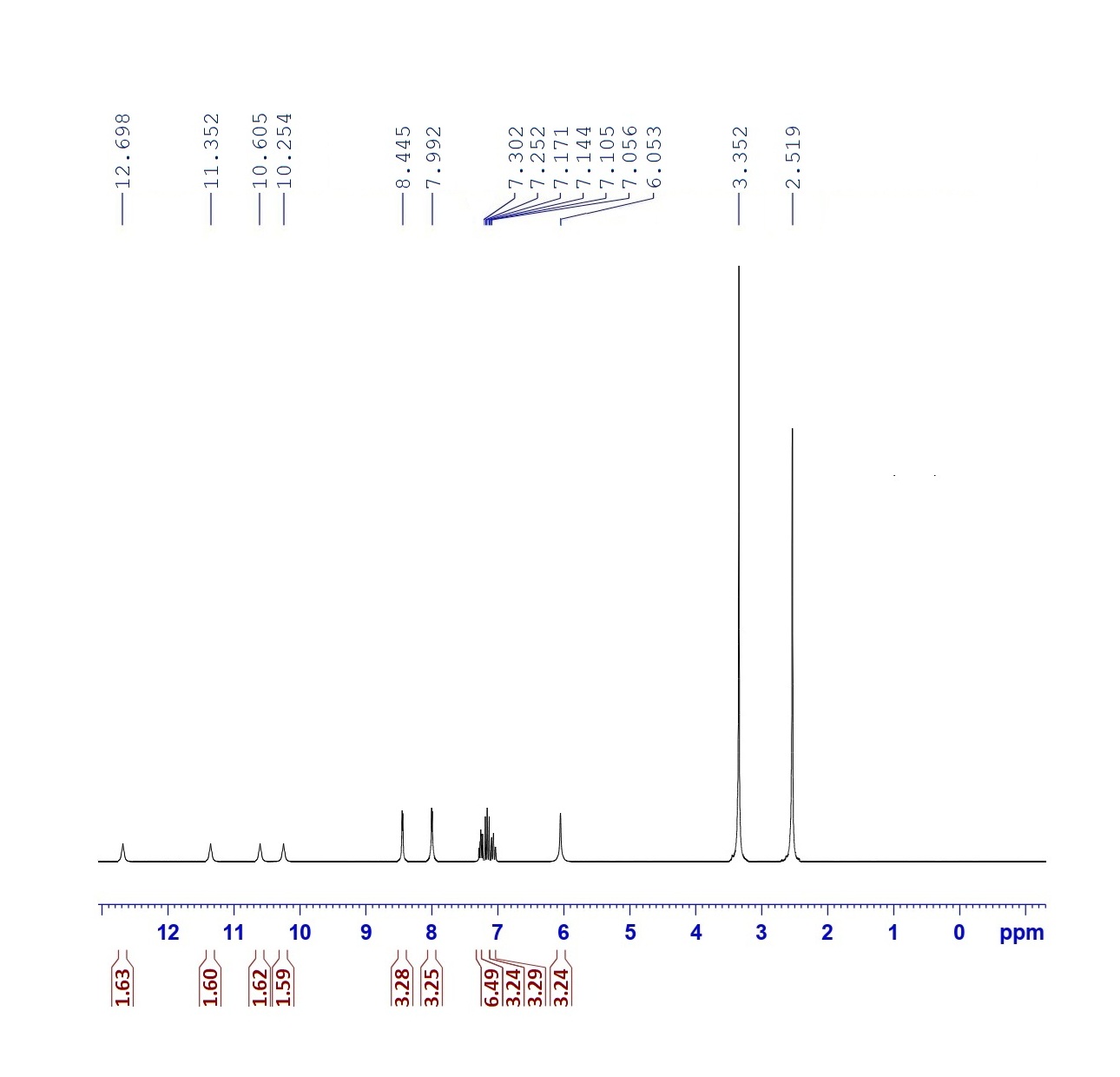
**

^1^H-NMR of compound **20**


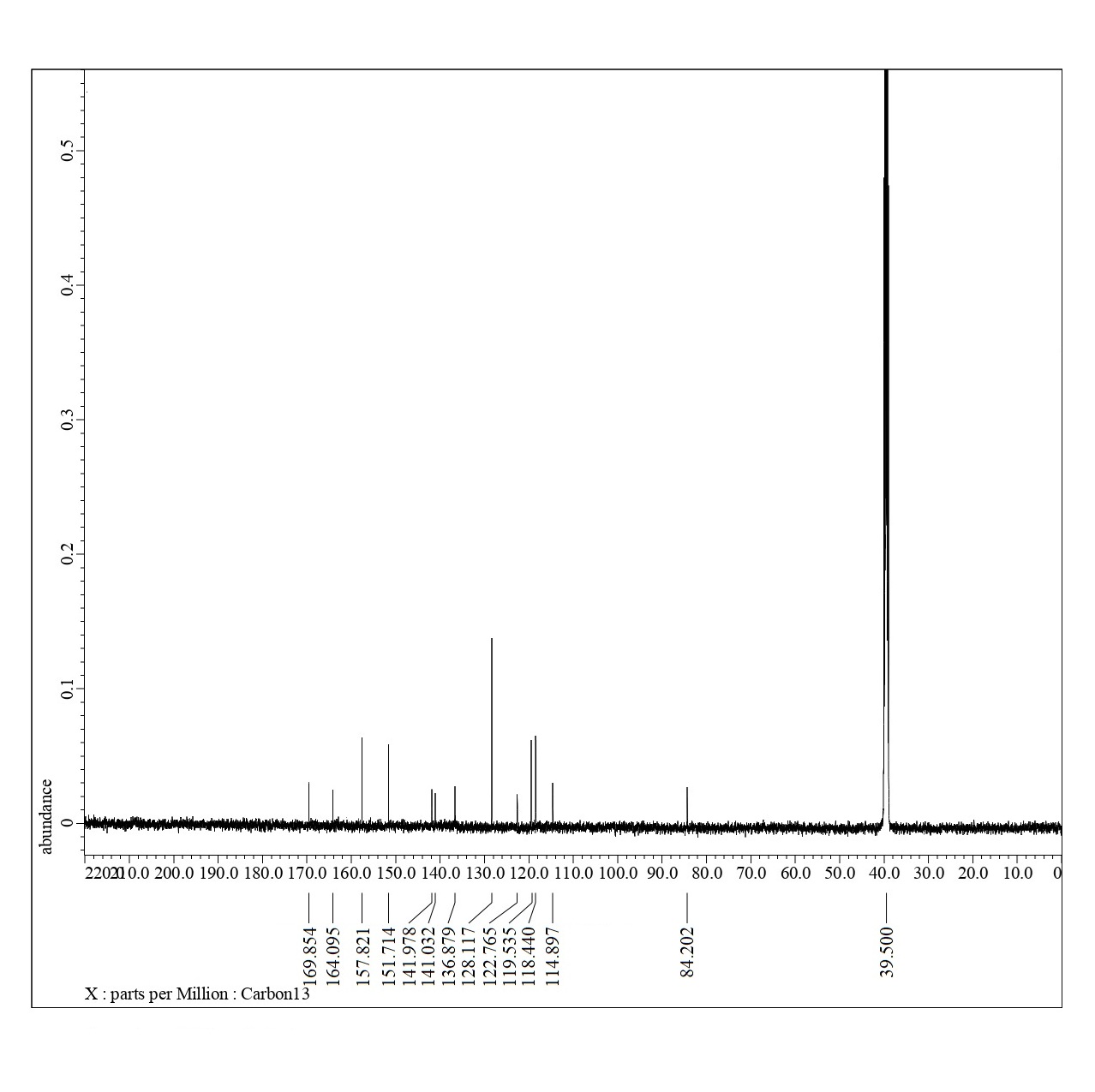


^13^C-NMR of compound **20**

**
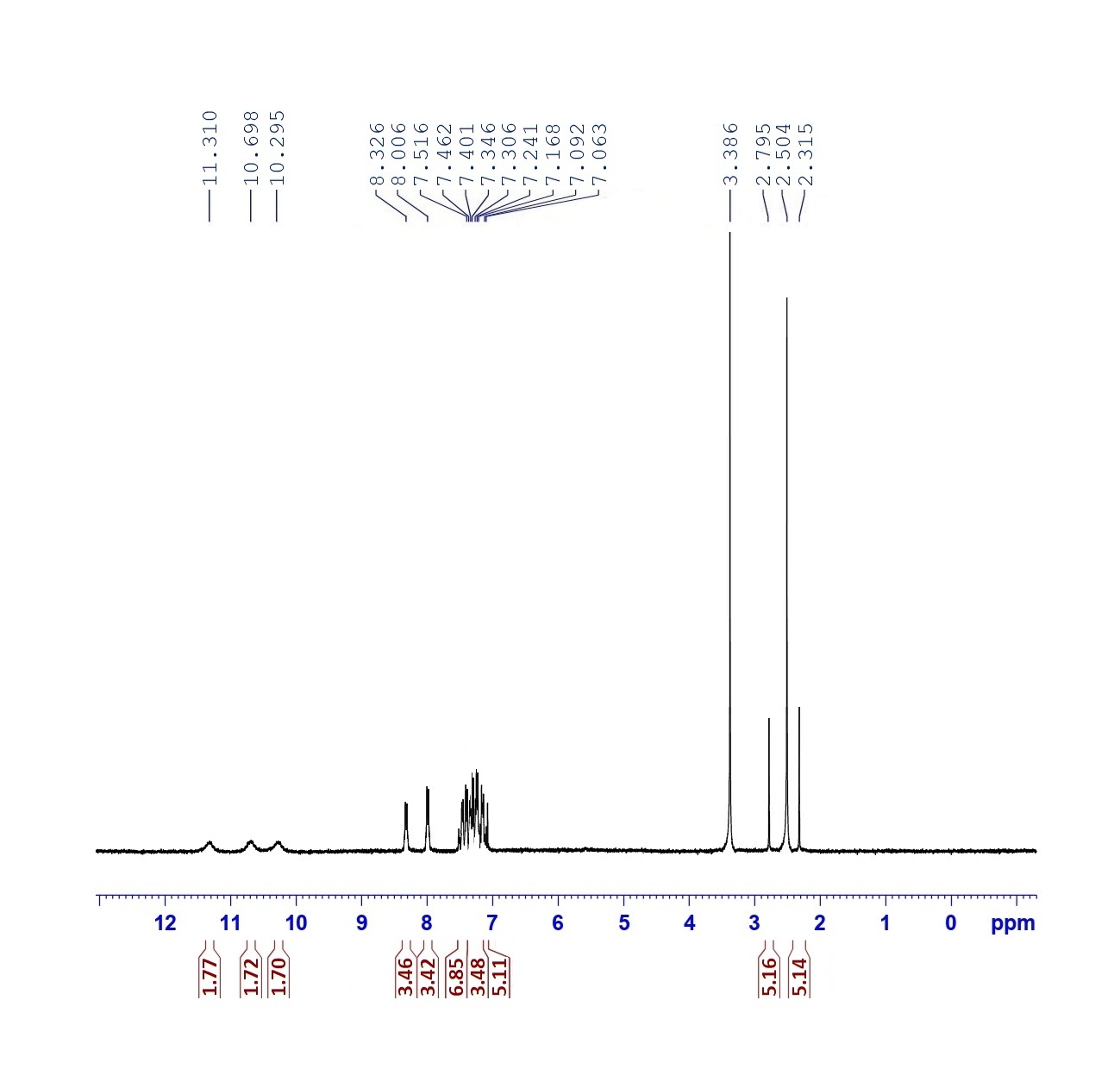
**

^1^H-NMR of compound **21**


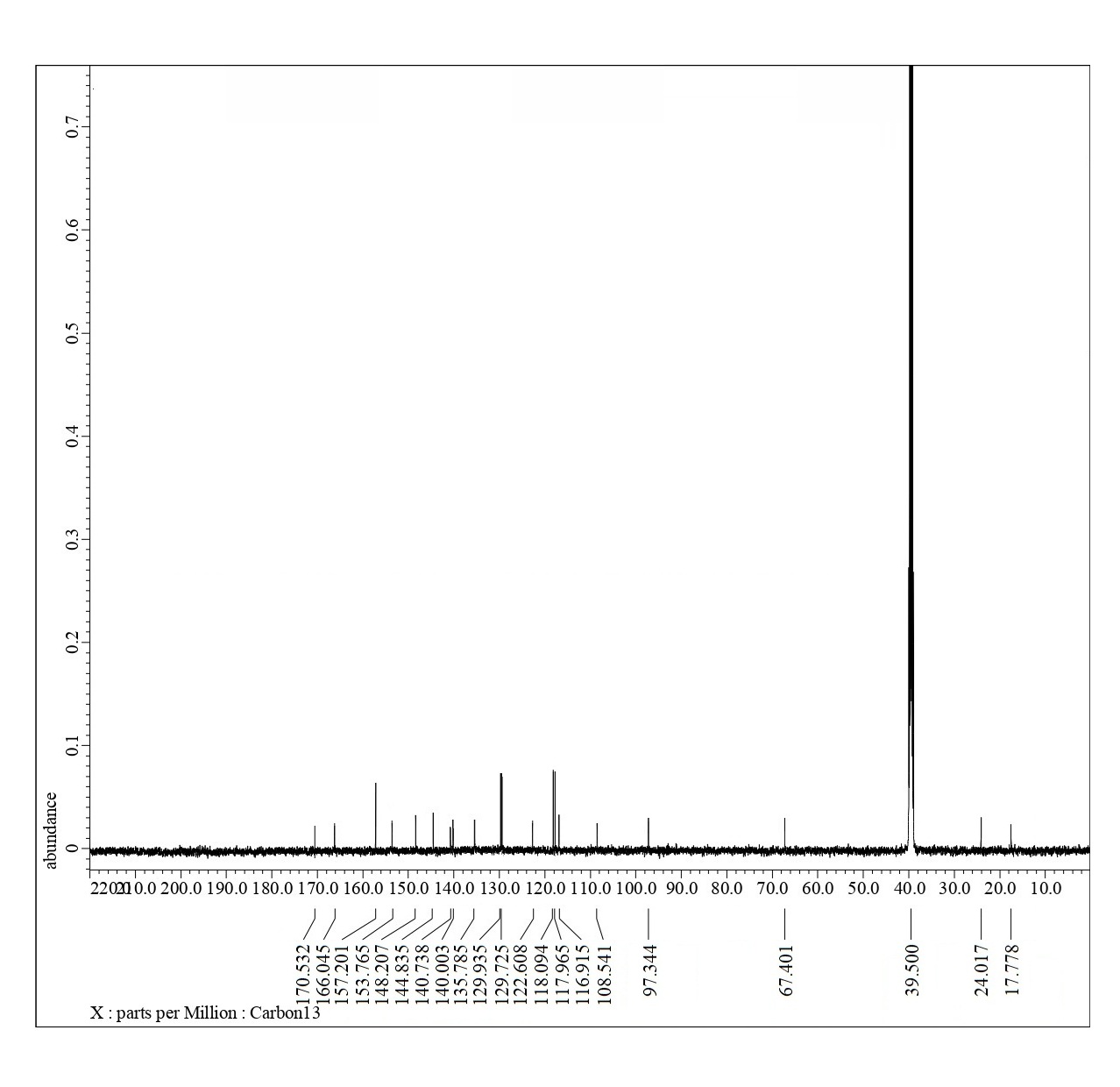


^13^C-NMR of compound **21**

**
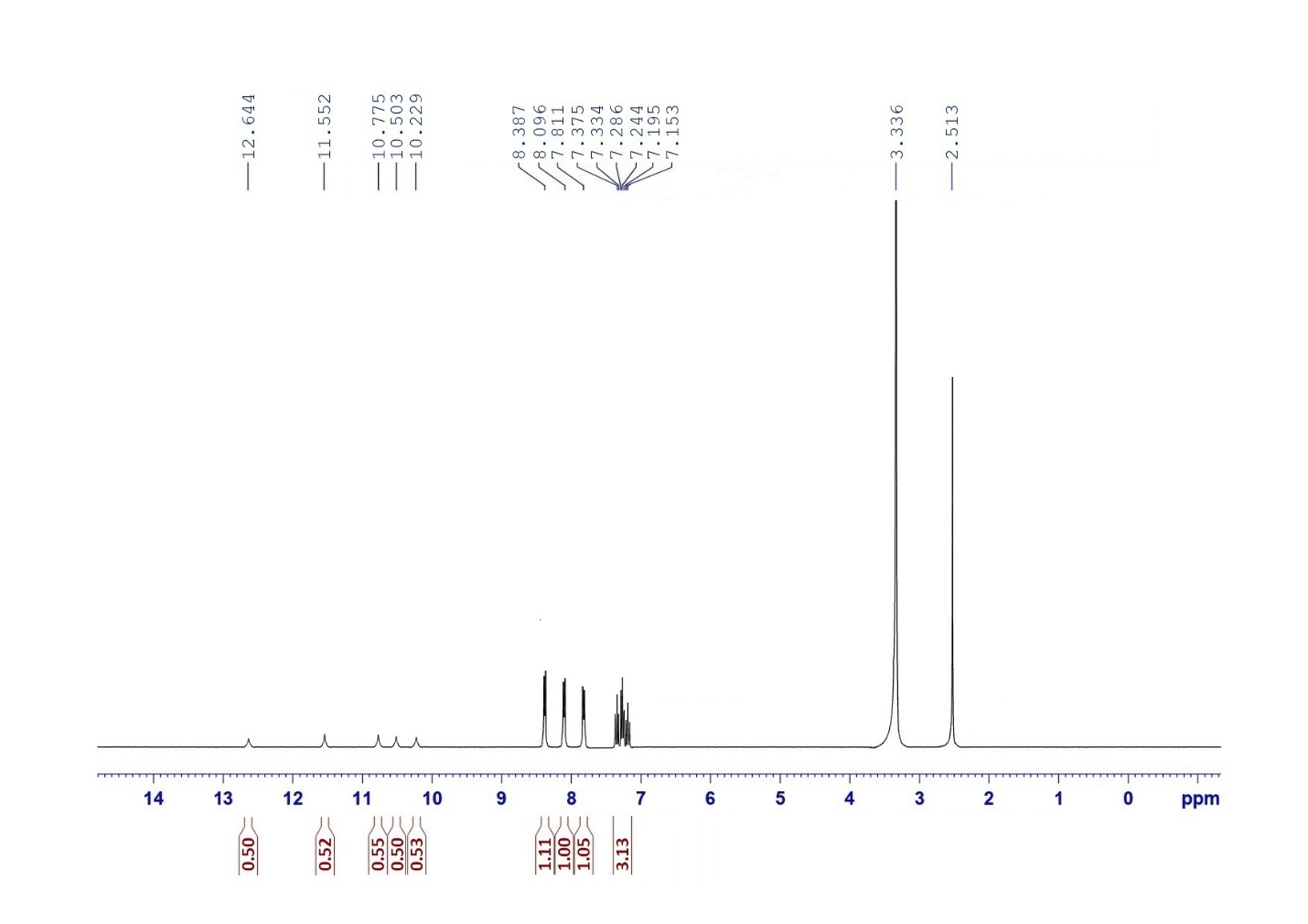
**

^1^H-NMR of compound **23**


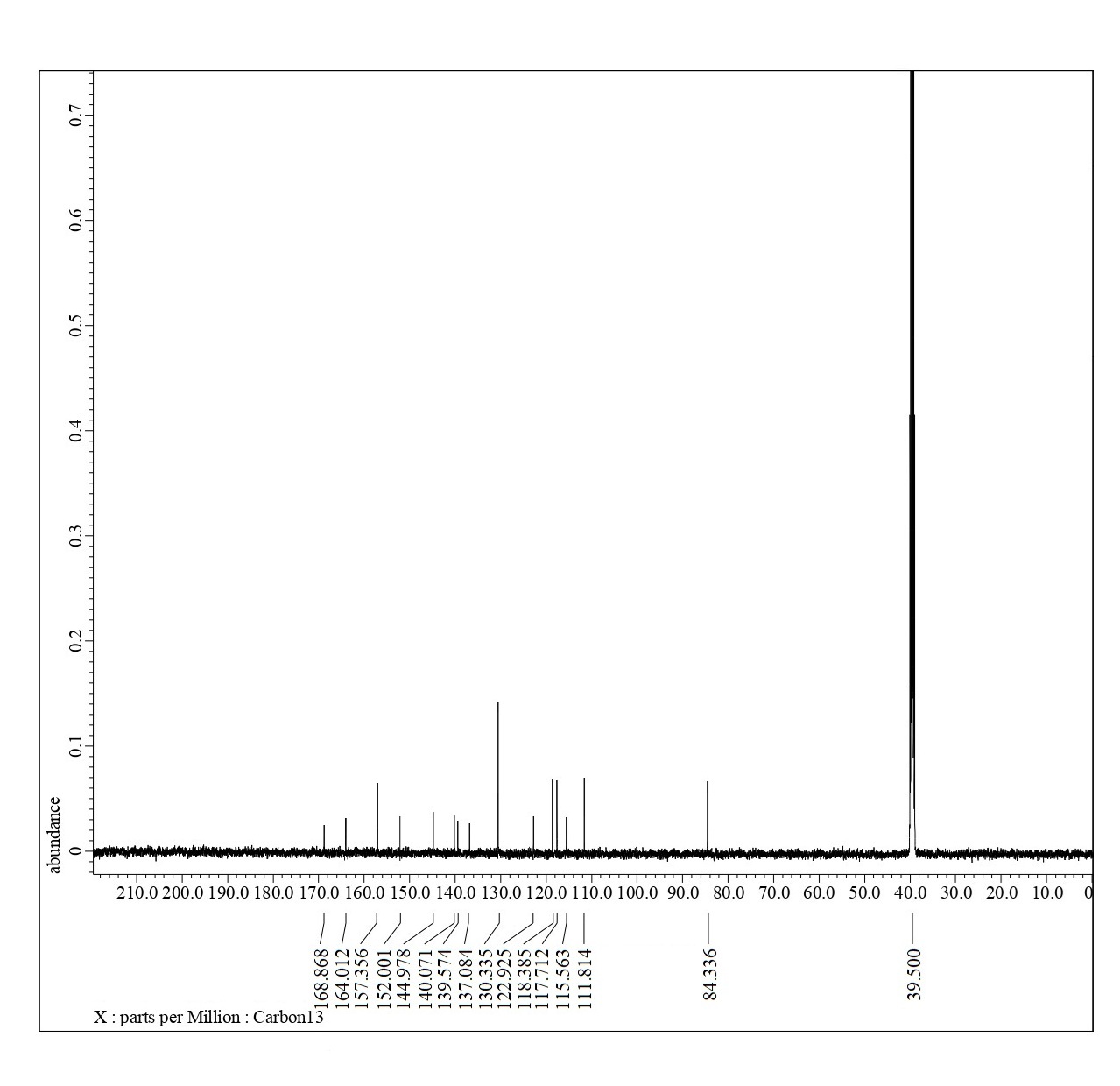


^13^C-NMR of compound **23**


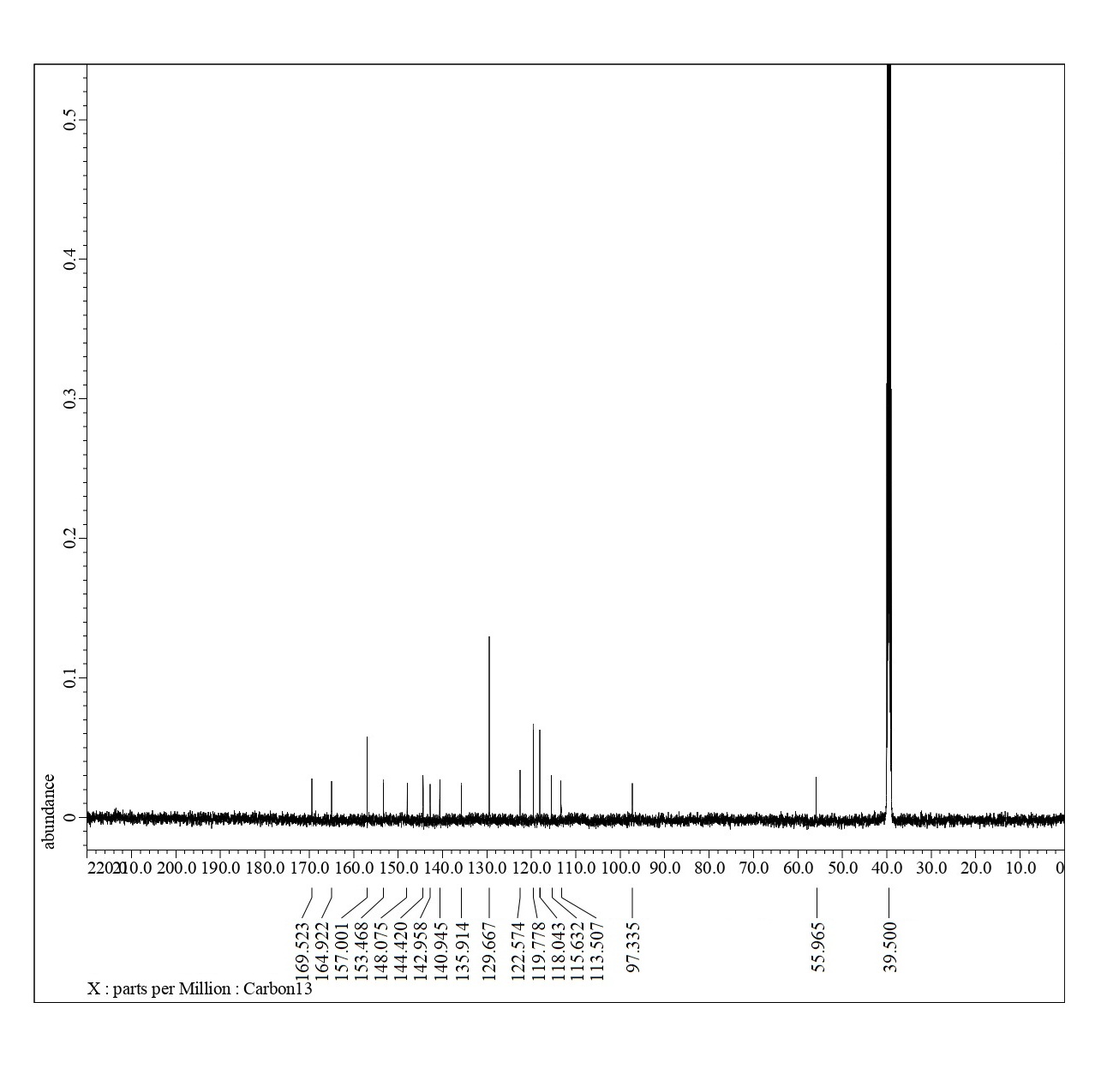


^13^C-NMR of compound **24**
